# The diversity of protein-protein interaction interfaces within T=3 icosahedral viral capsids

**DOI:** 10.3389/fmolb.2022.967877

**Published:** 2022-10-20

**Authors:** Digvijay Lalwani Prakash, Shachi Gosavi

**Affiliations:** Simons Centre for the Study of Living Machines, National Centre for Biological Sciences, Tata Institute of Fundamental Research, Bengaluru, India

**Keywords:** T=3 icosahedral viruses, coat-proteins, protein-protein interactions, dimerization interfaces, *Leviviridae*, *Bromoviridae*, *Tombusviridae*, *Tymoviridae*

## Abstract

Some non-enveloped virus capsids assemble from multiple copies of a single type of coat-protein (CP). The comparative energetics of the diverse CP-CP interfaces present in such capsids likely govern virus assembly-disassembly mechanisms. The T = 3 icosahedral capsids comprise 180 CP copies arranged about two-, three-, five- and six-fold axes of (quasi-)rotation symmetry. Structurally diverse CPs can assemble into T = 3 capsids. Specifically, the *Leviviridae* CPs are structurally distinct from the *Bromoviridae*, *Tombusviridae* and *Tymoviridae* CPs which fold into the classic “jelly-roll” fold. However, capsids from across the four families are known to disassemble into dimers. To understand whether the overall symmetry of the capsid or the structural details of the CP determine virus assembly-disassembly mechanisms, we analyze the different CP-CP interfaces that occur in the four virus families. Previous work studied protein homodimer interfaces using interface size (relative to the monomer) and hydrophobicity. Here, we analyze all CP-CP interfaces using these two parameters and find that the dimerization interface (present between two CPs congruent through a two-fold axis of rotation) has a larger relative size in the *Leviviridae* than in the other viruses. The relative sizes of the other *Leviviridae* interfaces and all the jelly-roll interfaces are similar. However, the dimerization interfaces across families have slightly higher hydrophobicity, potentially making them stronger than other interfaces. Finally, although the CP-monomers of the jelly-roll viruses are structurally similar, differences in their dimerization interfaces leads to varied dimer flexibility. Overall, differences in CP-structures may induce different modes of swelling and assembly-disassembly in the T = 3 viruses.

## Introduction

**FIGURE 1:**
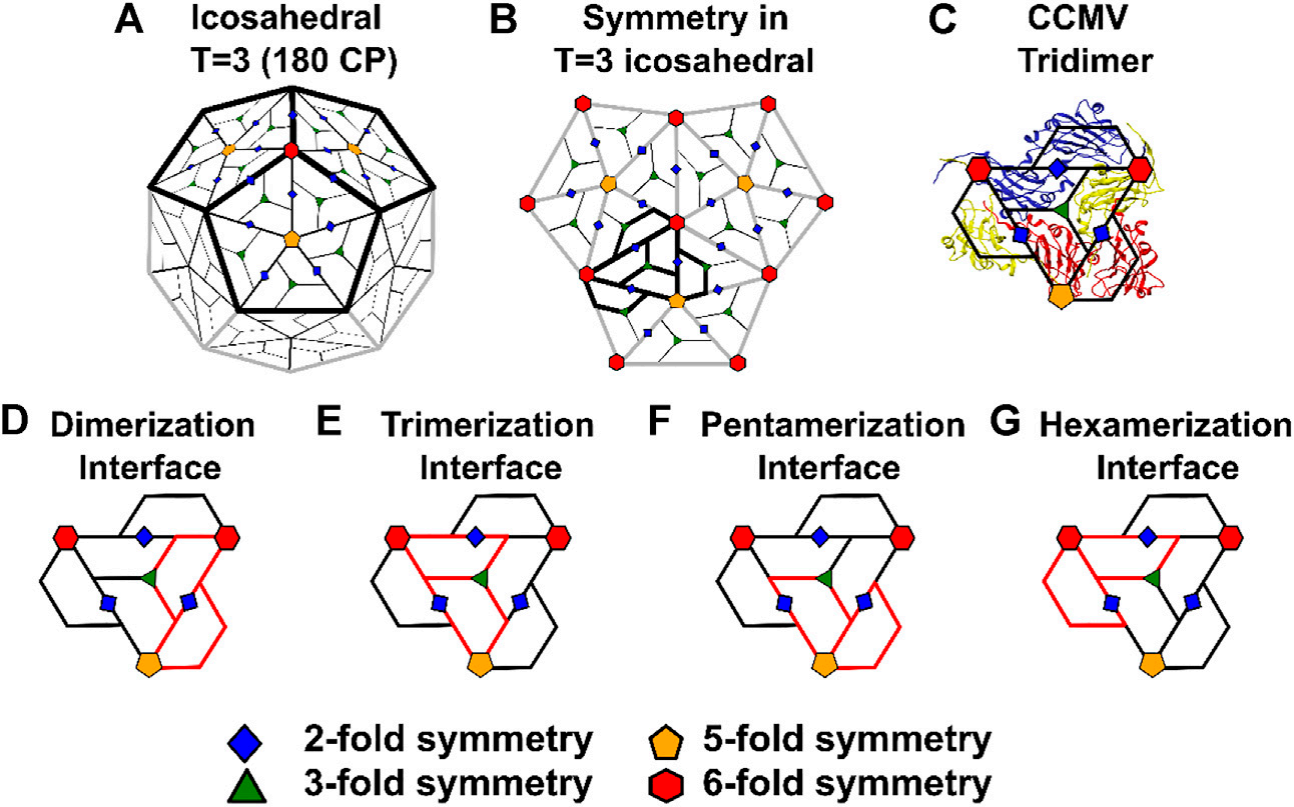
The arrangement of coat-proteins in T = 3 icosahedral viruses. A typical icosahedron consists of 20 equivalent equilateral triangular faces and 12 vertices. Each of the 12 vertices are shared by five faces, resulting in 12 five-fold axes of rotation symmetry (C_5_). There are two-fold rotation symmetry axes (C_2_) at the center of each of the 30 edges and a three-fold axis (C_3_) at the center of each of the five faces. In a T = 1 icosahedral capsid, each of the 20 triangular icosahedron faces are tiled by three equivalent coat-proteins (CPs). Thus, a T = 1 capsid has one CP in its icosahedral asymmetric unit (IAU), 20 × 3 = 60 CPs in total and exactly the same axes of rotation symmetry as the icosahedron. **(A)** A T = 3 capsid has three CP conformers (A, B, C) in its IAU leading to a capsid with 60 × 3 = 180 CPs. It is drawn here by placing a pentamer (three pentamers are marked with dark boundaries) around each C_5_ vertex (orange pentagon) of the original icosahedron. This pentamer is subdivided into 5 IAUs. The centers of the IAUs form 12 × 5 = 60 axes of quasi-three-fold rotation symmetry (QC_3_; green triangles). There are 30 C_2_ axes (originating from the icosahedron and between 2 C CPs; blue diamonds). Because there are three CPs per IAU, an additional 30 × 2 = 60 quasi-two-fold (QC_2_; blue diamonds) axes of rotation symmetry are formed between the A and B CPs of two adjacent IAUs belonging to the same pentameric unit. Finally, the original 20 C_3_ axes at the center of each face are converted into quasi-six-fold axes of rotation symmetry (QC_6_, red hexagons) due to the extra symmetry in the trimeric IAU. Thus, the T = 3 capsid consists of 20 QC_6_ (red hexagons), 12 C_5_ (orange pentagons), 60 QC_3_ (green triangles), 30 C_2_ (blue diamonds) and 60 QC_2_ (blue diamonds) axes. This representation scheme is preserved throughout the figures. **(B)** The three pentameric units marked with dark boundaries in **(A)** are enlarged and shown with all the axes of rotation symmetry marked. The region marked with dark boundaries shows the 6 CPs that form a tri-dimeric unit. This unit contains a central IAU flanked by three additional CPs belonging to adjacent IAUs. These three CPs are related to the CPs within the central IAU by (quasi-)two-fold rotation symmetry. Such tri-dimeric units contain every type of CP-CP interface that can be present in a T = 3 capsid. **(C)** The tri-dimeric structure from the capsid of Cowpea Chlorotic Mottle Virus (CCMV*, Bromoviridae*) with an overlay of the tri-dimeric unit from **(B)**. The three CP conformers from CCMV, A, B and C are colored red, yellow and blue respectively. For tri-dimer structures of representatives from all virus families, see Figure 2. **(D–G)** The various CP-CP interaction interfaces present in the tri-dimer. The two CPs which are part of the interaction are marked in red. The red line segment that is common to the two CPs represents the interaction interface. **(D)** The two CPs (CP2) which form the dimerization interface. There are three such interfaces in the tri-dimer. **(E)** The two CPs which form the trimerization interface. There are three such interfaces in the tri-dimer. **(F)** CPs which form the pentamerization interface **(G)** CPs which form the hexamerization interface. There are two such interfaces in the tri-dimer.

Non-enveloped viruses enclose their genome in a shell composed exclusively of proteins. In icosahedral viruses, this protein shell, also known as a capsid, has icosahedral symmetry (Louten, 2016). The capsids of some icosahedral viruses are assembled using a single type of protein, called the coat-protein (CP). Icosahedral viruses are further classified based on their triangulation (T) number, which, for simple viruses, is generally equal to the number of unique CP conformers that are present in the unit that is used to tile the icosahedron (Caspar and Klug, 1962). Thus, a T = 3 icosahedral capsid, composed of 180 CPs (Figure 1), has a CP trimer in each tiling unit (also known as the icosahedral asymmetric unit: IAU). Other distinct CP-CP interactions give rise to (quasi-)symmetric dimers, pentamers and hexamers (Figures 1A,B). Henceforth, the interface between the (quasi-)symmetric dimers (CP2) is called the dimerization interface, the interface between any two CPs from an IAU is called a trimerization interface and so on (Figures 1D–G).

The process of virus assembly is not completely understood even in viruses whose capsids are made of a single type of CP. Although proteins of the infected-host (HP) may affect virus assembly-disassembly, multiple experimental studies have used capsids expressed from bacterial expression systems, assembled in the absence of HP-CP interactions (Zhao et al., 1995; Sastri et al., 1999; Lokesh et al., 2002; Powell et al., 2012), or have studied disassembly with purified virus particles (Pappachan et al., 2009; Powell et al., 2012; Bond et al., 2020). In such assembly-disassembly processes, the balance of energetic interactions among CPs and between the CPs and the genome will determine the order of events in assembly. Capsids can also assemble around non-genome cargo (Ren et al., 2006; Sokullu et al., 2019; Durán-Meza et al., 2020) indicating that CP-CP interactions can by themselves direct capsid assembly. In order to understand potential principles that govern such CP-CP interface energetics, we analyzed the strength and nature of all CP-CP interfaces (Figures 1D–G) in diverse T = 3 icosahedral viruses.

**FIGURE 2:**
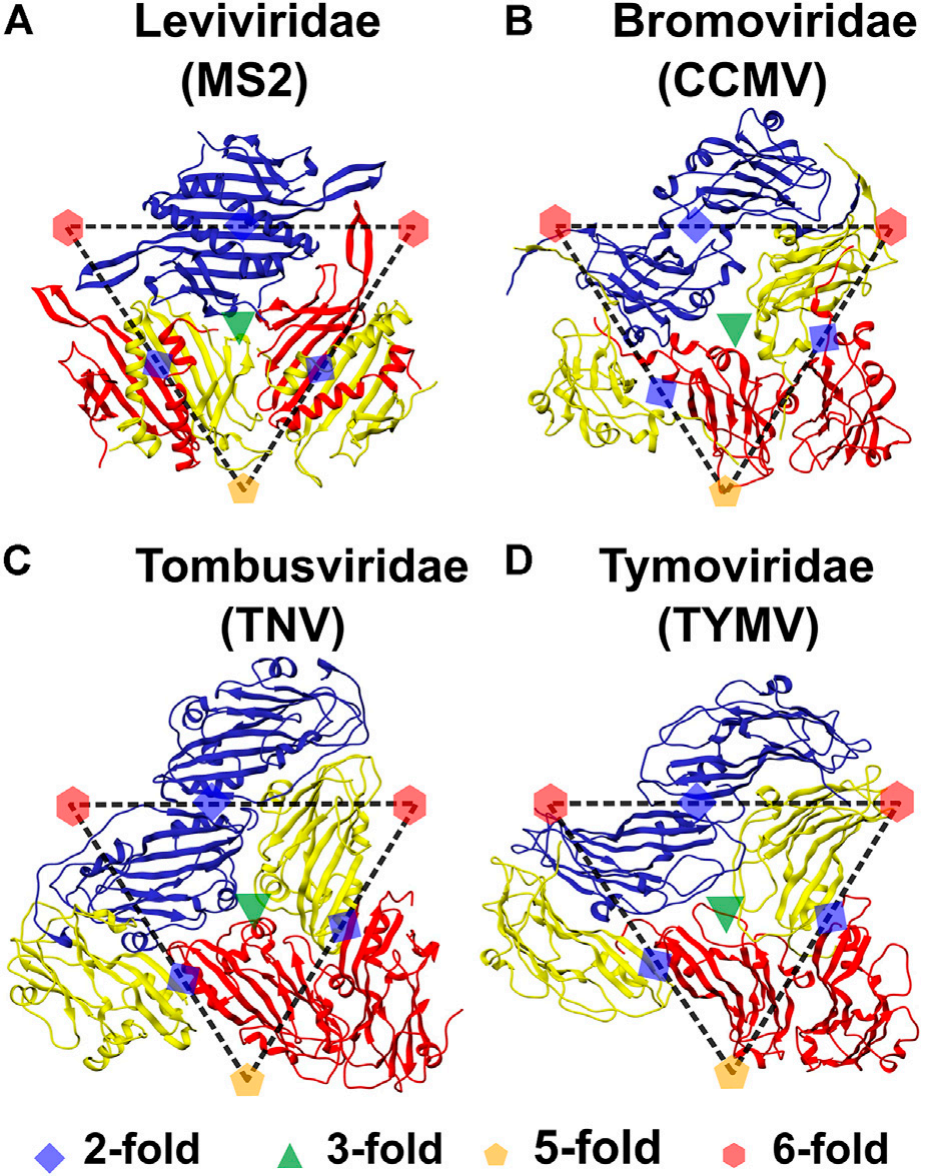
Symmetry elements in the tri-dimer structures of representatives from the four virus families. The structures are labeled by both the virus family and the virus name. All (quasi-)rotation symmetry axes can be shown using the dimers of the three monomers, termed A, B and C and approximately bounded by the dashed triangle, that form the Icosahedral Asymmetric Unit. In all four viruses, chains A, B and C are colored red, yellow and blue, respectively. The position of the axes of rotation symmetry are marked. The green triangle at the center of the IAU represents the QC_3_ symmetry axis and any pair of CPs within the IAU form the trimerization interface (Figure 1E). Red hexagons and orange pentagons represent QC_6_ and C_5_ symmetry axes respectively. Five A conformers are present around the C_5_ axes (Figures 1A,B) of which two are present in the tri-dimer and they form the pentamerization interface (Figure 1F). Alternating B and C conformers (three each) are present at the QC_6_ axes (Figures 1A,B) of which one B and one C conformer can be seen at each of the two QC_6_ axes and these form hexamerization interfaces (Figure 1G). A blue diamond between the two C CP chains (present in adjacent IAUs) indicates a C_2_ axis, while those between the A CP and B CP chains present in adjacent IAUs, indicate QC_2_ symmetry axes. The interactions between these dimers, termed CC CP2 and AB CP2 respectively, form the dimerization interface (Figure 1D). **(A)** Bacteriophage MS2 (MS2), **(B)** Cowpea Chlorotic Mottle Virus (CCMV; also see Figure 1C), **(C)** Tobacco Necrosis Virus (TNV) and **(D)** Turnip Yellow Mosaic Virus (TYMV). The chains (conformers) are named as in their PDB files. It should be noted that the A and B names are flipped in MS2 as compared to the jelly-roll viruses, with the B conformer being present in the pentamer and the A conformer in the hexamer. Tri-dimeric structures from each virus were used to calculate all the CP-CP interactions. Structures generated using UCSF Chimera (Pettersen et al., 2004).

The *Leviviridae*, *Bromoviridae*, *Tombusviridae* and *Tymoviridae* families have positive-sense single-stranded RNA genomes enclosed in T = 3 icosahedral capsids (King et al., 2012). However, while the bacteriophage *Leviviridae* CPs fold to an α+β fold with two α-helices and seven β-strands (Fold d.85 in SCOPe (Fox et al., 2014; Chandonia et al., 2022); Figure 2A), the *Bromoviridae*, *Tombusviridae* and *Tymoviridae* are mostly plant viruses whose CPs fold to the jelly-roll fold (SCOPe classification: b.121.4; Figures 2B–D). The jelly-roll fold is composed of two stacked four-stranded antiparallel β-sheets and is present in diverse virus CPs (Rossmann and Johnson, 1989; Johnson and Chiu, 2000; Cheng and Brooks, 2013). Despite the structural differences between the CPs, experimental disassembly data is available for at least one virus from each family which indicates that the viruses disassemble into dimers (Adolph and Butler, 1974; Stockley et al., 2007; Pappachan et al., 2009; Powell et al., 2012; Bond et al., 2020). The question that arises then is, does the overall capsid symmetry rather than interface energetics drive disassembly processes.

In the commonly studied *Levivirus* bacteriophage MS2, it is known that the capsid assembly unit is not CP but CP2, the homodimer composed of the two symmetry related CPs interacting at the dimerization interface (Ni et al., 1995; Stockley et al., 2007). Our earlier folding simulations (Prakash and Gosavi, 2021) showed that MS2-CP2 is an obligate dimer, i.e., it cannot fold into two independent fully folded monomers. MS2-CP2 also folds in a two-state manner, populating only the unfolded and dimeric folded ensembles without populating either a dimeric or a two-monomer intermediate ensemble. Using folding simulations, a previous study (Levy et al., 2004b) classified diverse homodimers into either two-state folding dimers or three-state folding dimers populating a partially folded ensemble. The authors argued that folding outcomes are probably determined by the nature of the dimeric interface and constructed two interface dependent parameters ad hoc. The first parameter (R), the ratio of the number of inter-monomer contacts to the intra-monomer contacts, is a proxy for the relative size of the interface. The second parameter (H), the average hydrophobicity of the residues in the interface, is a proxy for how buried (and not solvent exposed) the interface residues want to be. It was found that H and R could also classify homodimers into two-state (high H and R values) and three-state folders (lower H and R values).

We previously calculated the H and R for MS2-CP2 and these values predicted two-state folding in agreement with simulations (Prakash and Gosavi, 2021). Here, we hypothesize that H and R can also be more generally used to understand how similar CP-CP interaction interfaces are to each other. Accordingly, we calculated H and R for all the interfaces (dimerization, trimerization, pentamerization and hexamerization) of viruses from the four T = 3 families. We find that the dimerization interfaces of the *Leviviridae* have higher H and R values than all other interfaces indicating that these CP2 dimers are predicted to be two-state folders. The H values of the jelly-roll fold dimerization interfaces are generally marginally higher indicating that the interface residues may be buried more strongly through dimerization possibly enabling disassembly into CP2 dimers.

To further investigate the CP2 dimers, we performed molecular dynamics (MD) simulations of coarse-grained structure-based models (Clementi et al., 2000) of a representative CP2 from the four virus families. The inter-monomer distance changes the least in MS2-CP2 indicating a rigid interface while this distance varies the most in the *Bromoviridae* representative. Overall, differences in the structure of the dimerization interface give rise to distinct interface dynamics in the three CP2 jelly-roll fold dimers and this is likely to lead to different modes of assembly and disassembly.

## Methods

### Obtaining capsid structures for interface analysis

PDB IDs for T = 3 capsids from *Leviviridae*, *Bromoviridae, Tymoviridae* and *Tombusviridae*, were obtained from VIPERdb (http://viperdb.scripps.edu) (Montiel-Garcia et al., 2021) and filtered to eliminate redundant structures. Assembled capsid structures for the selected PDB IDs were then downloaded from RCSB PDB (Berman et al., 2000). From the complete capsid structures, a tri-dimer structure was extracted (Figure 2), which included one triangular IAU with A, B and C chains and one chain each extracted from three adjacent IAUs, such that these chains had a dimerization interface with the chains from the original IAU. This tri-dimer is the minimal unit containing all interactions between adjacent CPs in the T = 3 capsids (Figures 1C–G).

### Calculating normalized interface size and hydrophobicity

**FIGURE 3:**
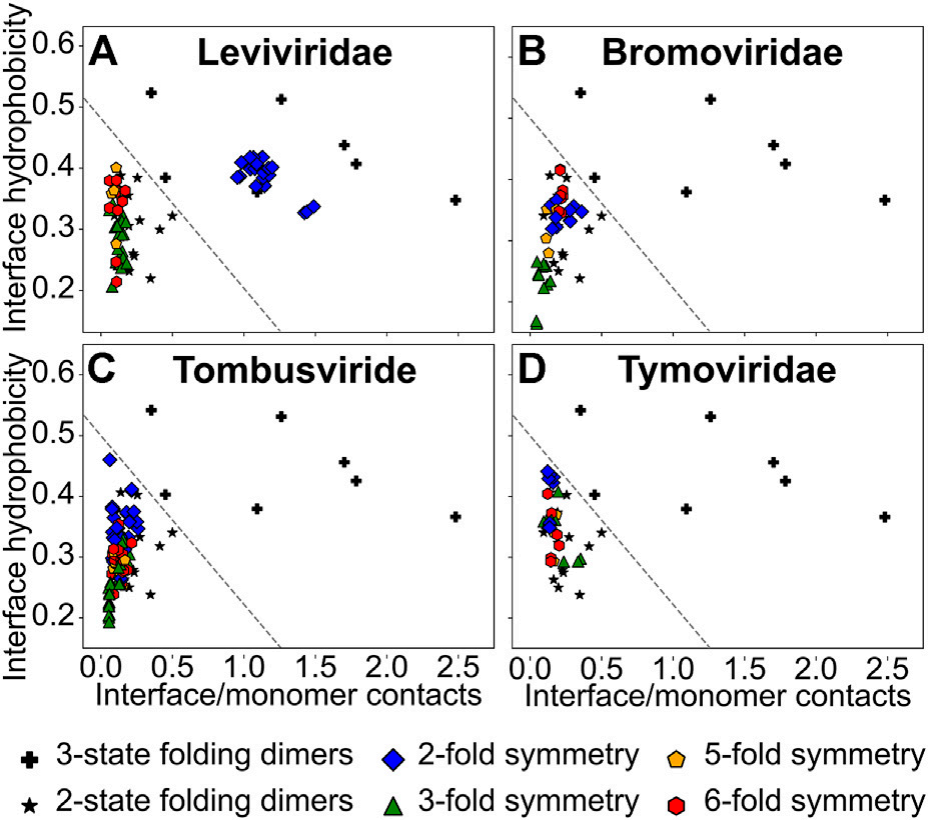
A plot of the normalized interface hydrophobicity (H) vs. the ratio of interface contacts to monomer contacts (R). **(A)** *Leviviridae*, **(B)** *Bromoviridae*, **(C)** *Tombusviridae* and **(D)** *Tymoviridae.* A three-state folding dimer (★) folds to an intermediate ensemble (either two monomers or a partially folded dimer) from two unfolded monomers before folding to the final dimeric state. A two-state folding dimer (+) folds directly to the dimeric state from two unfolded monomers. These datasets of homodimers are from a previous study on homodimer interfaces (Levy et al., 2004b) and are shown in all panels. An arbitrary line (y/0.5 + x/1.8 = 1), separating the two-state and the three-state dimers, is drawn to provide visual guidance (and does not represent an actual boundary between the two regions). The interfaces between two CPs related through the (quasi-)two-fold rotation symmetry (dimerization interface in CP2; blue diamonds), the quasi-three-fold rotation symmetry (trimerization interface; green triangles), the five-fold rotation symmetry (pentamerization interface; orange pentagons) and the quasi-six-fold rotation symmetry (hexamerization interface; red hexagons) are shown.

The H and R parameters (Figure 3) were calculated as done previously for diverse homodimers (Levy et al., 2004a; Levy et al., 2004b). When two residues are “close” in structure, they are said to be in contact. Here, interatomic intra-CP and inter-CP contacts were calculated using the CSU software (Sobolev et al., 1999) for every pair of adjacent CPs in the tri-dimer structure. These contacts were then projected onto their corresponding residues. For CPs labeled *x* and *y*, the number of intra-CP contacts are *N*_*intra(x)*_ and *N*_*intra(y)*_ respectively, while the inter-CP contacts are *N*_*inter(xy)*_. The ratio of the number of inter-CP to the intra-CP contacts is then

$$R_{\left(xy\right)}=\frac{N_{inter\left(xy\right)}}{{0.5N}_{intra\left(x\right)}+N_{intra\left(y\right)})}$$ (1)

Inter-CP interface hydrophobicity was calculated by averaging over the hydrophobicity factor, *h*_*i*_ (Pacios, 2001), for every residue *i*, which participated in an inter-CP contact. Thus,

$$H_{\left(xy\right)}=\overset{n}{\underset{i}{\sum }}\frac{h_{i}}{2N_{inter\left(xy\right)}}$$ (2)

where *n = 2×N*_*inter(xy)*_. It should be noted that if a residue participates in two different inter-CP contacts, then it is counted twice. *h*_*i*_ (and H) ranges from 0 to 1, with *h*_*i*_ = 1 for the most hydrophobic residue and *h*_*i*_ = 0 for the least hydrophobic residue. We calculate the hydrophobicity factors using a scale that was used in the original H parameter calculations for homodimers (Levy et al., 2004b).

### Multiple structure alignment of jelly-roll fold CPs

**FIGURE 4:**
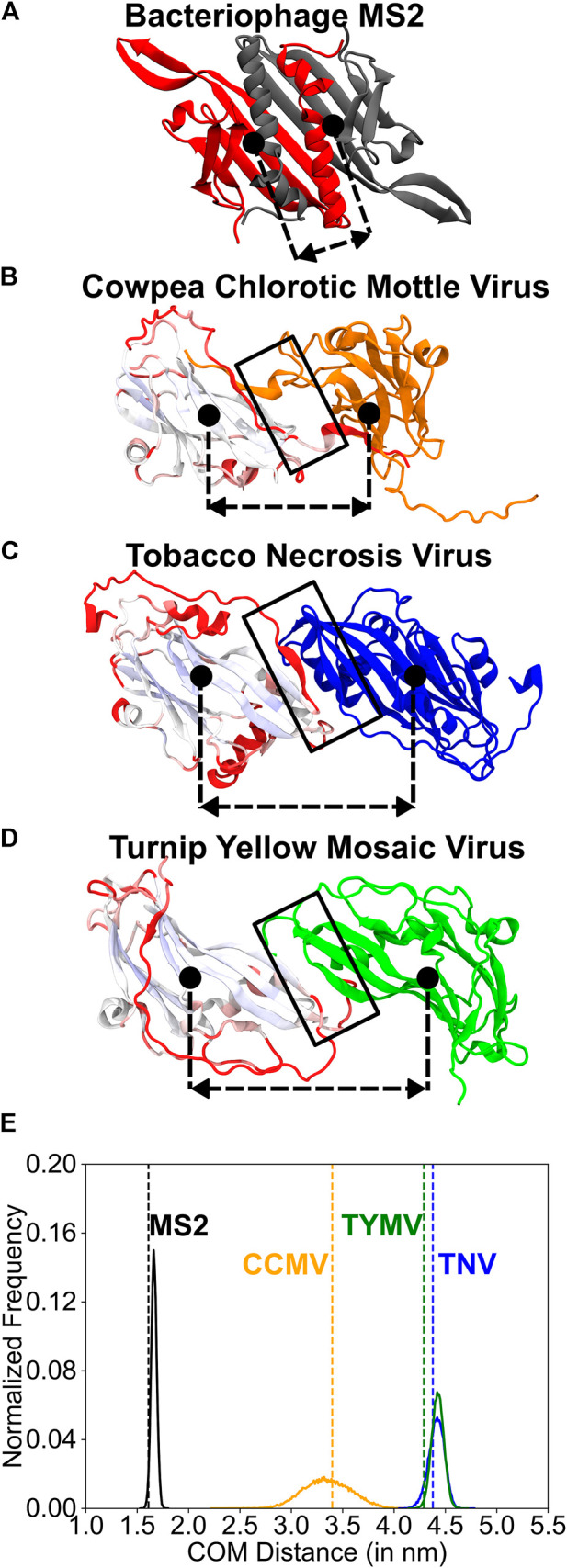
A comparison of the structure and the dynamics of the CP2 dimerization interfaces. **(A–D)** Structures of the two monomers related through two-fold rotation symmetry (dimerization interface). The dimerization interfaces are marked by a black rectangle. The approximate position of the center of mass of each protein chain is indicated using a filled black circle. The dashed-double sided arrow indicates the distance between these centers of mass (COM distance). The COM distance distribution is plotted in **(E)**. **(A)** MS2 (monomers in grey and red) **(B)** CCMV (graded and orange), **(C)** TNV (graded and blue) and **(D)** TYMV (graded and green). The monomers on the left in **(B–D)** were structurally aligned using the STAMP algorithm (Russell and Barton, 1992) within the Multiseq extension (Roberts et al., 2006) of the VMD package (Humphrey et al., 1996) and are shown in the aligned position. They are colored from blue through white to red based on how good the alignment is with blue indicating a good alignment and red indicating no alignment. The core β-sheet jelly-roll fold (pale blue to white) aligns well across the three viruses. The change in orientation in the right monomer (orange, blue and green) is due to the distinct dimer interfaces in the three viruses. **(E)** Histograms of the COM distance calculated from equal time coarse-grained simulations of the dimers. The vertical dashed line (colored like the histogram) shows the COM distance in the native folded structure. MS2-CP2: mean COM distance = 1.67 nm, standard deviation = 0.03 nm. TNV-CP2: mean = 4.42 nm, standard deviation = 0.08 nm. TYMV-CP2: mean = 4.43 nm, standard deviation = 0.06 nm. CCMV-CP2: mean = 3.35 nm, standard deviation = 0.23 nm. A broader histogram such as that seen in CCMV indicates a more flexible dimer interface. For each of these CP2 dimers, the alignment of the native structure with sampled structures whose COM distances are the mean, and mean ± 2×(standard deviation) are shown in Supplementary Figure S4.

Two ‘C’ CP monomers connected by a two-fold rotation symmetry (CC-CP2) were extracted from the tri-dimer structures of representative viruses (Figure 2): *Bromoviridae*: Cowpea Chlorotic Mottle Virus: CCMV (PDB ID: 1CWP); *Tombusviridae*: Tobacco Necrosis Virus: TNV (PDB ID: 1C8N); and *Tymoviridae*: Turnip Yellow Mosaic Virus: TYMV (PDB ID: 1AUY). For one monomer from each of the CP2s, we performed a multiple structure alignment using the STAMP algorithm (Russell and Barton, 1992) as implemented in the Multiseq extension (Roberts et al., 2006) of the VMD package (Humphrey et al., 1996). The aligned CPs (Figures 4B–D left chains) were colored based on their alignment from blue through white to red, with well-aligned regions in blue and unaligned regions in red. The second chains (Figures 4B–D right chains) of the dimers were not aligned. These jelly-roll CP2s and the MS2-CP2 (also extracted from the bacteriophage MS2 tri-dimer; PDB ID: 2MS2, Figure 4A) were used to understand dimer dynamics.

### Simulating CP2 dynamics

We used a structure-based model coarse-grained to a single Cα-bead per residue (Cα-SBM) (Clementi et al., 2000) to simulate one CC-CP2 dimer (see previous section) of a representative virus from each family. This Cα-SBM was previously used to simulate the folding of MS2-CP2 and its variants using MD simulations (Prakash and Gosavi, 2021). Details of the Cα-SBM and simulations are described in the supplementary information. The distance between the centers of mass (COM) of the two chains in the dimer was calculated for structures sampled during a simulation. These COM distance values were then binned (bin width 0.01 nm) into a histogram (distribution) which gives the normalized number of structures present in the bin with a given COM distance value.

## Results and discussion

### The *Leviviridae* CPs are obligate dimers

As stated in the introduction, two parameters, R, the ratio of interface to monomer contacts, and H, the average interface hydrophobicity, were previously shown to be able to classify a set of diverse homodimers as being two-state or three-state folding in simulations (Levy et al., 2004b). Since R and H are physically intuitive interface properties with R being a proxy for interface size and H for its “stickiness”, we use these parameters more generally to understand the strength and nature of CP-CP interfaces.

We first calculated the H and R values of the set of homodimers used previously (Levy et al., 2004b) and drew an arbitrary line in the H-R space such that homodimers which lie on one side of the line are two-state folders while those on the other side are three state folders (Figure 3). However, it should be underlined that this division is arbitrary and there are insufficient data points to demarcate the boundary exactly. So, homodimers that lie near the boundary-line, on either side, could either be two-state with a small folding barrier or three-state with a weakly populated intermediate. Since we intend to use H and R more generally here, we assume that homodimers that lie in the two-state region have stronger interfaces than homodimers that lie near the boundary which in turn have stronger interfaces than homodimers that lie in the three-state folding region.

Our previous folding simulations of the *Levivirus* MS2-CP2 agreed with the H and R values and showed that MS2-CP2 was a two-state dimer indicating that MS2-CP2 has an extensive interface which couples the folding of the two CPs (Prakash and Gosavi, 2021). Other *Leviviridae* CP2 also have high H and R values (Figure 3A) indicating that CP2 is the capsid assembly unit in the entire family. In contrast, all other *Leviviridae* CP-CP interfaces (the trimerization, pentamerization and hexamerization interfaces) have lower R values. Further, trimerization interfaces are the least hydrophobic (Figure 3A).

An examination of MS2-CP2 shows a two-layer interface (Figure 4A). The inner layer is formed by the anti-parallel β-β hydrogen bonding interactions between the edge β-strands (named βG in MS2) of the two extended β-sheets, one from each CP. The outer layer comprises the C-terminal α-helices of each CP stacked over the β-sheet of the partner CP. The CP helices are also sandwiched between the partner CP’s N-terminal β-hairpin and C-terminal α-helices, creating an interdigitated topology (Figure 4A). This two-layer structure gives rise to an extensive interface area with R varying between 0.95–1.50. Moreover, 50–70% residues participating in interface contacts are non-polar, giving rise to large H values of 0.35–0.45 (Supplementary Table S1). Previously (Prakash and Gosavi, 2021), we had computationally designed two MS2-CP2 variants, each having reduced interactions in one of the interface layers. One variant was a pseudo-circular permutant (CiP) CP2 which had the C-terminal helices from each monomer excised and linked to the N-terminus of the partner monomer (Supplementary Figure S1A). This preserved all dimer contacts but converted inter-CP β-sheet-helix contacts into intra-CP contacts. The H and R values of CiP-CP2 lie close to the line dividing the two-state and three-state folders (Supplementary Figure S1B). In agreement, CiP-CP2 is a two-state folder with a barrier much smaller than that of wild type (WT) MS2-CP2. Further unlike WT-MS2-CP, CiP-CP monomers can fold completely (Prakash and Gosavi, 2021). A similar effect (two-state folding with a low barrier and high H values with lower R values) was seen in the other variant of MS2-CP2 (termed MS2-ΔβG:βG-CP2) in which the interactions between the two antiparallel βG edge strands were deleted.

In summary, for each of the CP-CP interfaces (dimerization, trimerization, pentamerization and hexamerization), the H and R values are similar across *Leviviridae*. Additionally, the *Leviviridae* have intertwined dimerization interfaces which lead to higher R values and two-state folding of MS2-CP2. We next analyze the CP-CP interfaces of the other three families and compare these with those of MS2-CP2.

### The CP2 interfaces of the jelly-roll fold viruses are similar to their other interfaces

The sizes of the dimerization interfaces (CP2; Figures 2B–D) of the CPs from the three jelly-roll virus families are similar to their corresponding pentamerization and hexamerization interfaces with R ranging from 0.07 to 0.50 (Supplementary Tables S2–S4). However, except in *Bromoviridae* (Figure 3B, Supplementary Table S2), the dimerization interface is generally marginally more hydrophobic than the pentamerization and hexamerization interfaces (Figures 3C,D, Supplementary Tables S3, S4). The trimerization interface in *Bromoviridae* and *Tombusviridae* is the least hydrophobic (Figures 3B,C) while in *Tymoviridae*, the trimerization interface is closer in hydrophobicity to the pentamerization and hexamerization interfaces (Figure 3D, Supplementary Table S4). Overall the H and R values of all the CP-CP interfaces of the jelly-roll viruses are also similar to the non-CP2 (non-dimerization) interfaces of the *Leviviridae*.

What then could be the reason for CP2 either forming first or persisting after capsid disassembly in disassembly/reassembly experiments with jelly-roll fold viruses (Adolph and Butler, 1974; Zhao et al., 1995; Sastri et al., 1999; Zlotnick et al., 2000; Tang et al., 2006; Powell et al., 2012) as well as in experiments involving assembly defective mutants (Tang et al., 2006; Pappachan et al., 2009)? We find that although the H and R values for all the CP-CP interfaces of the jelly-roll fold families lie in the three-state folding region, the dimerization interfaces lie close to the arbitrary line (dashed-line in Figures 3A–D) separating this region from the two-state folders. Thus, the interface of CP2 is likely to be stronger than interfaces that lie in the three-state folding region and is likely to remain intact in conditions where the other interfaces break, leading to early formation of and late dissociation of CP2 dimers in assembly/disassembly experiments. Consequently, we focus on the CP2 interfaces and examine them in detail.

### Despite having structurally-similar monomers, the dimerization interfaces of the jelly-roll fold viruses are distinct

Since the three jelly-roll fold virus families have structurally similar CPs, we performed a multiple structure alignment on one monomer from the CP2 of one representative virus from each of the three families (see Methods; Figures 4B–D). The core β-sheet region forming the jelly-roll fold is fairly well-aligned in all three virus CPs, but each CP has additional secondary structure elements and a completely different dimerization interface (Figures 4B–D). The TNV and TYMV CP2s have an interface involving side-by-side interactions between the edge β-strands of the two CPs (Figures 4C,D), reminiscent of the antiparallel β-sheet inner layer of MS2-CP2 (Figure 4A). In addition to these β-β interactions, the TNV-CP2 interface also contains small α-helices from each monomer stacked over its own β-sheet that are also packed against each other (Figure 4C). This makes the interface similar to that of MS2-CiP-CP2 (Supplementary Figure S1A) and as expected, their H and R values are also similar (Supplementary Figure S1B).

The CCMV-CP2 interface is distinct from the TYMV and TNV CP2 interfaces and lacks β-β interactions. Each monomer has a C-terminal tail which reaches out and interacts with the β-sheet of the core jelly-roll fold of the partner monomer (Figure 4B). This is similar to the variant, MS2-ΔβG:βG-CP2, in which the interchain β-β contacts were deleted (Prakash and Gosavi, 2021). The role of the swapped C-terminal helices in MS2-ΔβG:βG-CP2 is played by the swapped C-terminal tails in CCMV. The lack of β-β contacts and the length of the C-terminal tails creates a space or a cavity between the two CPs of the CCMV-CP2 (Supplementary Figure S2B) and this gap should make the CCMV-CP2 interface (and that of other *Bromoviridae*) more flexible.

To test this hypothesis, we simulated the CP2s from MS2, CCMV, TNV and TYMV using Cα-SBMs and calculated the distributions of the distance between the centers of mass of the two monomers (COM distance) from the simulation trajectory (see Methods). As expected, the two-layer MS2-CP2 interface has the most sharply peaked distribution (Figure 4E, Supplementary Figure S3) with the least standard deviation from the mean COM distance. The stiffness of this interface is pictorially depicted in Supplementary Figure S4A. TNV-CP2 and TYMV-CP2 have distributions which are more similar to each other (Figure 4E, Supplementary Figures S4C,D) than they are to CCMV-CP2. CCMV-CP2 (Figure 4E) has a broad COM distance distribution with the highest standard deviation. This flexibility in the CCMV-CP2 dimerization interface (Supplementary Figure S4B) has been shown experimentally and computationally (Tama and Brooks, 2002; Elrad and Hagan, 2008) to allow capsids with varying sizes. Capsids from *Bromoviridae* have also been shown to swell at neutral pH = 7.0 and at low divalent metal ion (Ca^2+^ or Mg^2+^) concentrations (Speir et al., 1995). The modelled pseudoatomic structure of the swollen capsid (Tama and Brooks, 2002) retains the pentamerization and hexamerization interfaces but a change in conformation at the dimerization interface expands the pore at the trimerization axis (QC_3_, Figure 2). Thus, *Bromoviridae* CP2 flexibility may be responsible for the ability of the capsid to swell. An additional role of the capsid flexibly could be to allow it to package genomes of slightly varying sizes. Unlike *Tymoviridae* and *Tombusviridae*, viruses belonging to *Bromoviridae* have their genome divided in four parts that are packaged separately in three different capsids (tripartite genomes) (Chaturvedi and Rao, 2018; Chakravarty and Rao, 2021). It was also shown that the C-terminal tail which forms the dimerization interface in *Bromoviridae* may be important for cell-to-cell movement of the viral capsids (Okinaka et al., 2001). In fact, *Bromoviridae* reassembly studies have shown aggregates other than regular T = 3 or T = 1 capsids (Bancroft et al., 1967; Adolph and Butler, 1974; Tang et al., 2006).

### Capsid asssembly and disassembly for understanding infections and in protein engineering

Due to the size and strength of the *Leviviridae* dimerization interfaces, they are expected to be stronger than the trimerization, hexamerization and pentamerization interfaces, implying that during disassembly, CP2-CP2 interactions will break before interactions within a CP2 dimer (intra-CP2), as was shown in previous acid disassembly experiments (Stockley et al., 2007). In contrast, because of the relative chemical similarity between the dimerization, pentamerization and hexamerization interfaces in jelly-roll viruses, these interactions are likely to break at similar denaturant conditions, while the trimerization interfaces may break in milder conditions (Szoverfi and Fejer, 2022). The swelling of the CCMV capsid by increasing the size of the pore at the trimerization axis (Speir et al., 1995; Tama and Brooks, 2002) is in accordance with this observation. A previous computational study (Polles et al., 2013) determining the rigid mechanical units in virus capsids, showed that while CP2 was the mechanically stable unit in MS2, pentamers and hexamers were stable in CCMV. Although the pentamers and hexamers are mechanically rigid units, the swapped C-terminal tails in CCMV-CP2 link hexamers to both adjacent hexamers and pentamers potentially buffering the breaking apart of viruses during disassembly.

The difference in the protein interfaces may also enable different infection mechanisms. *Leviviridae* infection is believed to involve disassociation of the maturation protein (which replaces one CC-CP2 in the WT virus) along with the genomic RNA from the capsid (Meng et al., 2019). In contrast, for *Bromoviridae*, infection is hypothesized to follow co-translational disassembly, where the genomic RNA is exposed for translation due to swelling of the capsid at the neutral pH of the host cell (Roenhorst et al., 1989; Speir et al., 1995; Tama and Brooks, 2002; Bond et al., 2020). Interestingly, similar swelling is also observed in both *Tombusviridae* (Aramayo et al., 2005; Llauró et al., 2015) and *Tymoviridae* (Virudachalam et al., 1985; Sastri et al., 1999), which do not have flexible CP2 interfaces. Thus, although it is assumed that they too co-translationally disassemble like *Bromoviridae*, the rates of swelling and infection could be very different.

Overall, even coarse-grained parameters and models which do not contain atomic details of the interfaces can be used to both understand and propose hypotheses in assembly-disassembly mechanisms especially when such mechanisms are studied *in vitro*. However, it is important to note that the present coarse-grained approaches do not include metal ions, virus RNA or host proteins and RNA. For instance, ordered genomic RNA such as is present in *Leviviridae* (Koning et al., 2016; Rolfsson et al., 2016; Chang et al., 2022) can also modulate the mechanism of viral assembly by coding specific sites for RNA-protein interactions called packaging signals (ElSawy et al., 2010; Stockley et al., 2016; Martín Garrido et al., 2020). Packaging signals are also present in *Bromoviridae* (Choi et al., 2002; Choi and Rao, 2003; Chakraborty et al., 2018), *Tombusviridae* (Qu and Morris, 1997) and *Tymoviridae* (Bink et al., 2003; Larson et al., 2005) although it is known that the RNA in *Bromoviridae* is much less ordered (Beren et al., 2020). We do not consider the effect of these signals here.

Finally, we discuss possible applications of understanding CP-CP interactions for capsid engineering. MS2 (*Leviviridae*) capsids have been used to design vessels for delivering therapeutic molecules (Wu et al., 1995; Brown et al., 2002; Wu et al., 2005; Galaway and Stockley, 2013; Yan et al., 2014; Pumpens et al., 2016) and for antigen presentation in vaccines (Mastico et al., 1993; Wu et al., 1995; Peabody, 1997; Tumban et al., 2015; Frietze et al., 2016; Pumpens et al., 2016). Similar studies have also been performed with T = 3 jelly-roll capsids (Ren et al., 2006; Barnhill et al., 2007; Lockney et al., 2011; Yildiz et al., 2012; Zeng et al., 2013; Sokullu et al., 2019; Durán-Meza et al., 2020). Understanding protein-protein interactions is an important step in improvising such systems, as was shown in a recent study on MS2 (Biela et al., 2022), where insertions were made to tweak the dimerization interface, allowing the formation of larger capsid structures.

## Conclusions

The capsids of the *Leviviridae*, *Bromoviridae*, *Tymoviridae* and *Tombusviridae* families have T = 3 icosahedral structures made up, almost completely, of a single type of coat-protein (CP). However, the structures of the *Leviviridae* CPs are distinct from those of the CPs of the other three virus families which fold into the jelly-roll fold. We examined the average hydrophobicity (H) and relative size (R) of all the CP-CP interfaces (dimerization, trimerization, pentamerization and hexamerization) and found that the dimerization interface of the *Leviviridae* CPs is distinctly different from all other interfaces with high H and R values indicating that these CPs are likely to be present as CP2 dimers. On an average, H values of the dimerization interfaces of the other three families (*Bromoviridae*, *Tymoviridae* and *Tombusviridae*) are also higher than that of the other interfaces potentially making this interface stronger. This could be the reason for capsid disassembly into CP2 in *in vitro* experiments. Although the CP monomers from these three virus families are structurally similar, differences in dimer interface structure make the dynamics of the dimerization interfaces distinct across the three families. Thus, even in viruses with the same overall capsid symmetry, a diversity in CP structure and interactions may lead to diverse assembly-disassembly mechanisms.

## Data Availability

The raw data supporting the conclusions of this article will be made available by the authors, without undue reservation.
