# Supplementary material for: The diversity of protein-protein interaction interfaces within T=3 icosahedral viral capsids
Source: Front Mol Biosci. 2022 Oct 20;9:967877. doi: 10.3389/fmolb.2022.967877 (PMC9631432; doi:10.3389/fmolb.2022.967877)
Supplement: Supplementary file 1 [file DataSheet1.PDF]

Supplementary Material

for

The diversity of protein-protein interaction interfaces within T=3 icosahedral viral capsids.

Digvijay Lalwani Prakash[1] and Shachi Gosavi[1,*]

[1]Simons Centre for the Study of Living Machines, National Centre for Biological Sciences, Tata Institute of Fundamental Research, Bengaluru 560065, India

*Correspondence to:

Shachi Gosavi (shachi@ncbs.res.in)

Phone: +91-80-23666105

SUPPLEMENTARY METHODS

Calculating the percentages of hydrophobic residues in interface contacts. For CPs labeled *x* and *y*, the number of inter-CP (interface) contacts is $N_{inter}(x,y)$. The number of residues that participate in such contacts is then $n(x,y)=2\times N_{inter}(x,y)$. The number of aromatic (WFY) and non-polar aliphatic (GAVLIM) residues that participate in these inter-CP contacts was counted and is termed $n_{HP}(x,y)$. The percentage of hydrophobic interface residues is given by $P(x,y)=n_{HP}(x,y)/n(x,y)\times 100$ (Tables S1-S4).

Structure Based Models (Cα-SBM). Structure-based models (SBMs), originally developed to study protein folding (Onuchic et al., 1997; Onuchic and Wolynes, 2004), encode the protein structure in their potential energy functions through attractive backbone (bond and angle), dihedral angle (secondary structure) and contact (tertiary structure) terms whose minima are determined from the native structure of the protein(s). This simplifies the potential energy function and permits the extensive sampling of the long time scale motion involved in protein folding and conformational dynamics using molecular dynamics (MD) simulations. In order to understand the dynamics of the dimerization interface, we used a common structure-based model (SBM) coarse-grained to a single Cα bead per residue (Cα-SBM) (Clementi et al., 2000) to construct the potential energy function of the CC CP2 dimers (see Methods). This Cα-SBM was previously used to simulate the folding of the MS2-CP2 dimers using molecular dynamics (MD) simulations (Prakash and Gosavi, 2021). Its potential energy function is as follows:

$$E_{bond} = \sum_{bond} K_b(x-x_0)^2 + \sum_{angle} K_a(\theta-\theta_0)^2 + \sum_{dihedral} K_d[(1-\cos(\phi-\phi_0)+0.5(1-\cos(3(\phi-\phi_0))] \qquad \text{Eq. S1}$$

$$E_{nonbond} = \sum_{\substack{native \\ contacts \\ i,j>i+3}} K_c \left[5\left(\frac{\sigma_{ij}}{r_{ij}}\right)^{12} - 6\left(\frac{\sigma_{ij}}{r_{ij}}\right)^{10} \right] + \sum_{\substack{non \\ contact \\ i,j<i+3}} K_r \left(\frac{C_{ij}}{r_{ij}}\right)^{12} \qquad \text{Eq. S2}$$

$$E = E_{bond} + E_{nonbond} \qquad \text{Eq. S3}$$

The protein is represented as a chain of beads with a bead positioned at the Cα atom of every residue. Attractive bonds (Eq. S1) are present between every pair of adjacent beads (*i, i+1*) with the minimum of the bond potential, x_0, being the distance between the same beads in the native structure of the protein. Similarly, the minima of the angle (θ_0) and the dihedral angle (ϕ_0) potentials are also calculated from the native structure as the angle (*i, i+1, i+2,*) and the dihedral angle (*i, i+1, i+2, i+3*) between the beads shown in brackets. Only those beads which are in "contact" have an attractive interaction (first term in Eq. S2) between them. The minimum of this attractive non-bonded interaction, the contact distance (σ_{ij}) is the distance between the (*i, j*) beads in the native structure. All beads which are not in contact interact through the second term in Eq. S2 with $C_{ij}=0.4\ nm$. The strengths of the bond ($K_b=100\varepsilon$) and angle ($K_a=20\varepsilon$) potentials (ε is the basic energy scale of the potential energy) constrains the bonds and angles to be close to their value in the native structure. The strengths of the dihedral angle ($K_d=1\varepsilon$) and the non-bonded potentials ($K_c=K_r=1\varepsilon$) allow these interactions to break. ε is set to $1\ kJ\ mol^{-1}$ and all distances are calculated in *nm*.

The same contacts used to calculate R (see Methods; determined from the protein structure using the CSU software (Sobolev et al., 1999)) were used as native contacts in the Cα-SBM. Input files for the simulations were generated using the SMOG server (Noel et al., 2016) and MD simulations using these input files were performed using GROMACS (van der Spoel et al., 2005) version 4.5.4. Simulations were performed for $5X10^7$ steps of 0.0005 ps each at 100 K. As can be

seen from its potential energy function, the Cα-SBM is a simplified model with implicit solvation, no Coulomb-like long range interactions and ad hoc energy terms which are defined using the natural energy scale of GROMACS ($\varepsilon = 1\ kJ\ mol^{-1}$). Since the temperature is set by the kinetic energy of this simplified model, it cannot be directly compared with the temperatures in experiments. We chose a temperature for all CP2 simulations which was lower than the simulation temperature at which MS2-CP2 unfolds (Prakash and Gosavi, 2021). This ensured that the CP2 remained folded during the simulations and motion due to the flexibility of the interface could be isolated. Here, we provide the value of the simulation temperature only to aid reproducibility.

The Cα-SBM assigns equal masses to all Cα beads. The center of mass (COM) distance was calculated using the GROMACS analysis program *g_dist* between the centers of masses of the two simulated chains in the dimer structure. Sampled structures (25000 structures equally spaced across the simulation trajectory) were binned based on the COM distance between the chains with a bin width of 0.01 nm and the distribution was normalized by dividing the number of structures in every bin by the total number of sampled structures.

SUPPLEMENTARY FIGURES

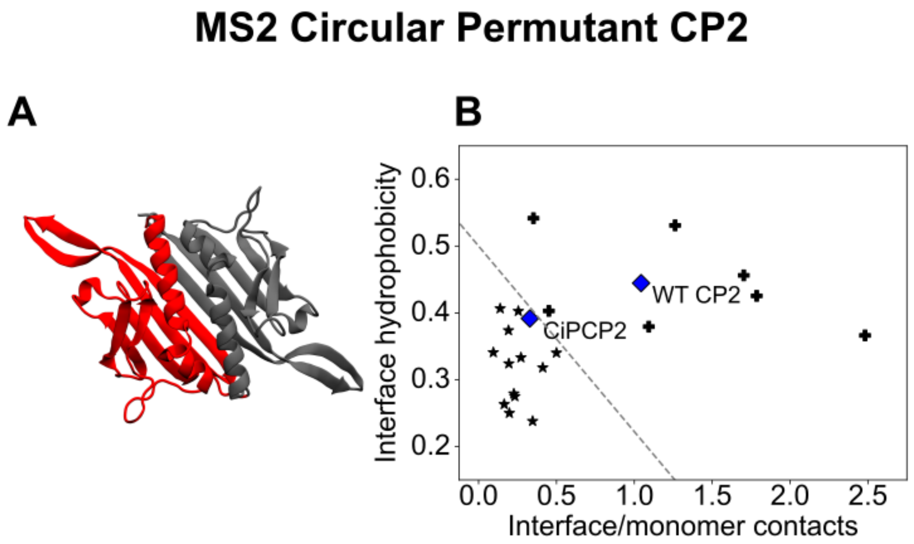

Figure S1. (A) Structure of the designed Circular Permutant variant of MS2 coat protein CC dimer (CiP-CC-CP2). Unlike in wild-type (WT) MS2-CP2 (Fig. S2A for instance), the helix-sheet interactions are intra-monomer in CiP-CP2. (B) A plot of interface hydrophobicity (H) versus ratio of interface to intra-monomer contacts (R) shows the two state folding dimers (+) and three state folding dimers (★) separated by a dashed line (x/1.8+y/0.5=1) as in Fig. 3. The line is arbitrary and only for visual guidance. The (R, H) values of the WT-CP2 (R=1.044, H=0.445) (PDB: 1ZDH) and CiP-CP2 (R=0.329, H=0.392) dimer interface are marked (blue diamonds). Circular permutation reduces the number of inter-protein contacts and in turn, the size of the interface and R. This brings the position of CiP-CP2 close to the arbitrary line separating the two-state and the three-state folding dimers. The (R, H) values of the MS2-ΔβG:βG-CP2 are (R=0.887, H=0.470). A similar figure is shown in the supplementary information of previous work (Prakash and Gosavi, 2021) which also provides further details about the folding dynamics of the CiP-CP monomer and the CiP-CP2 dimer. All structure figures in the supplementary information were made using UCSF-Chimera (Pettersen et al., 2004).

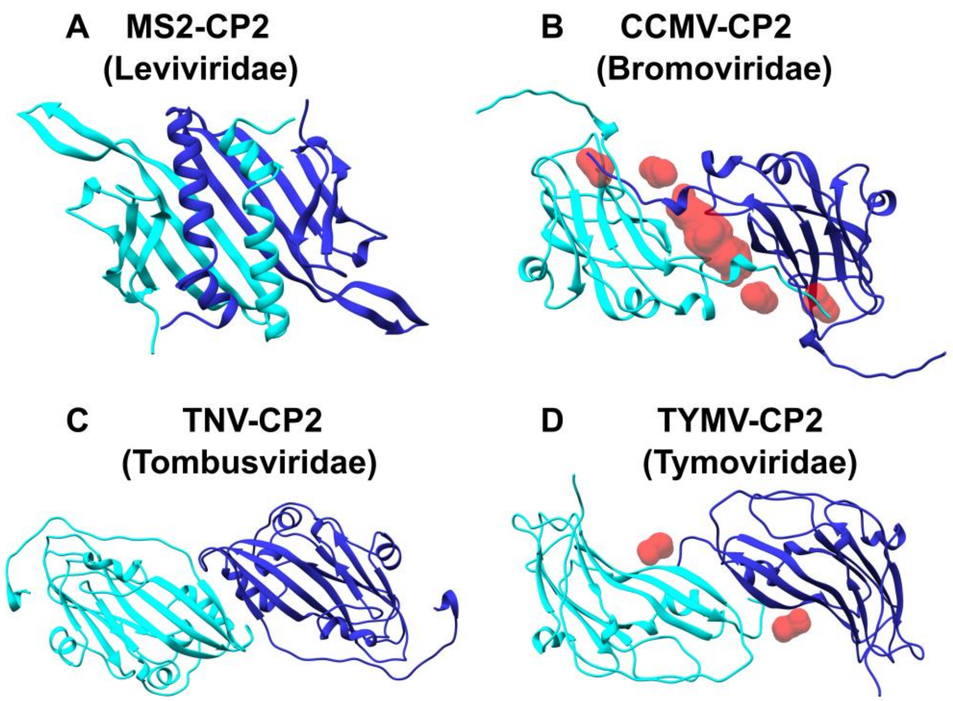

Figure S2. The two monomers of CCMV-CP2 seemed well-separated visually. This observation was confirmed by calculating cavities near the dimerization interface in CP2. (A) Bacteriophage MS2, (B) Cowpea chlorotic mottle virus (CCMV), (C) Tobacco necrosis virus (TNV) and (D) Turnip yellow mosaic virus (TYMV). Cavities were predicted using the fpocket tool (https://github.com/Discngine/fpocket) (le Guilloux et al., 2009) with a minimum probing sphere size of 0.4 nm. A large cavity is seen between the two monomers of CCMV-CP2 because this interface is held together only by the long C-terminal loop of one CP reaching out and interacting with the β-sheet of the other CP. In contrast, due to the side-by-side packing of helices and β-strands of the two monomers, no cavity was observed at or near the dimerization interfaces of MS2-CP2 and TNV-CP2 and only Small cavities were observed in TYMV-CP2 at the edges of the dimerization interface.

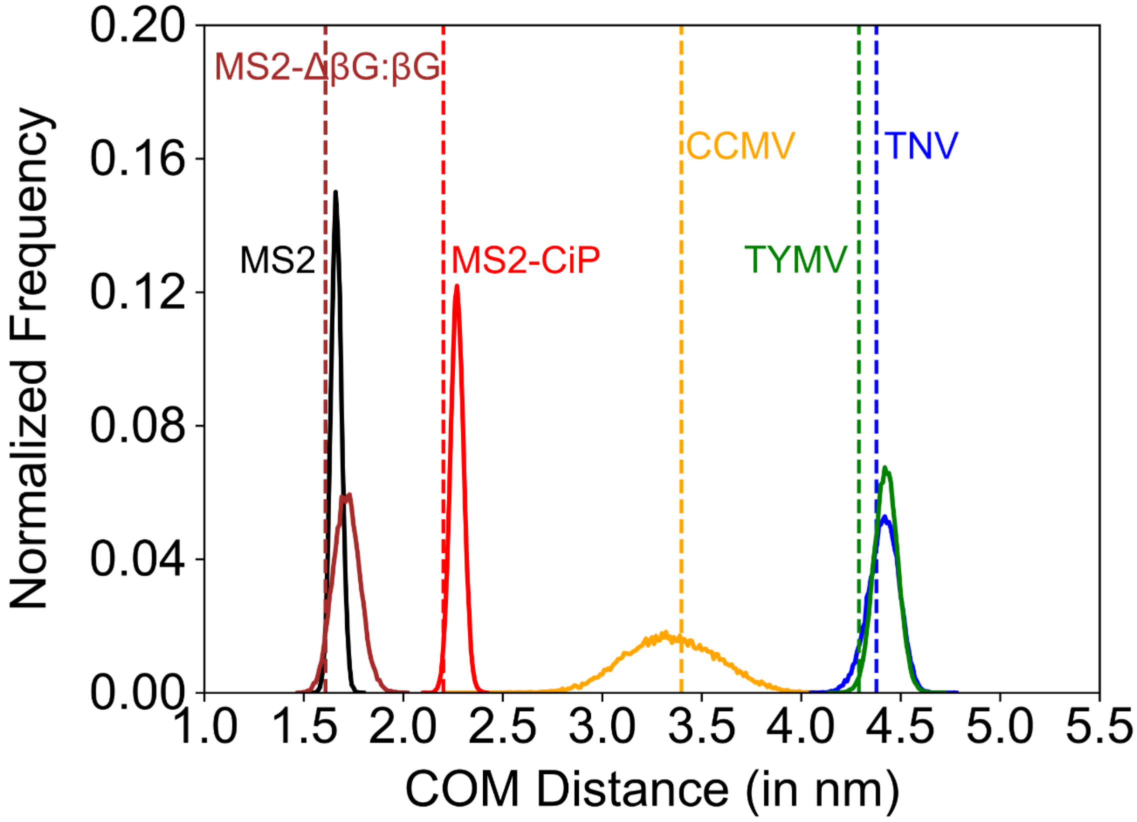

Figure S3. A histogram of the center of mass (COM) distance calculated from equal time coarse-grained simulations of CP2 dimers. The dashed line with the same color as the histogram shows the COM distance in the native folded structure. In addition to the histograms plotted in Fig. 4E (MS2 (black), CCMV (orange), TNV (blue) and TYMV (green)), the histograms of the two MS2 variants: circular permutant (CiP)-CP2 (red; mean at 2.28 nm with a standard deviation of 0.03 nm) and MS2-CP2 with deleted inter-chain βG-βG contacts (MS2-ΔβG:βG-CP2; brown; mean at 1.72 nm with a standard deviation of 0.07 nm) are also plotted. Circular permutation creates a less intertwined interface in MS2-CiP-CP2 than in MS2-CP2 (compare Fig. S1A with Fig. S2A) and this moves the centers of mass away from the interface, increases the COM distance and makes the COM distribution a slightly broader. The MS2-CiP-CP2 interface is similar to the TYMV and TNV interfaces (Fig. S2C and S2D). Due to the deletion in contacts, the MS2-ΔβG:βG-CP2 has no interactions between the interface β-strands similar to the CCMV-CP2 (Fig. S2B). This makes the interface flexible and the COM distribution is broader. The COM distance in the native structure is the same for MS2-CP2 and MS2-ΔβG:βG-CP2, their dashed vertical lines overlap and the black line for MS2-CP2 is not visible.

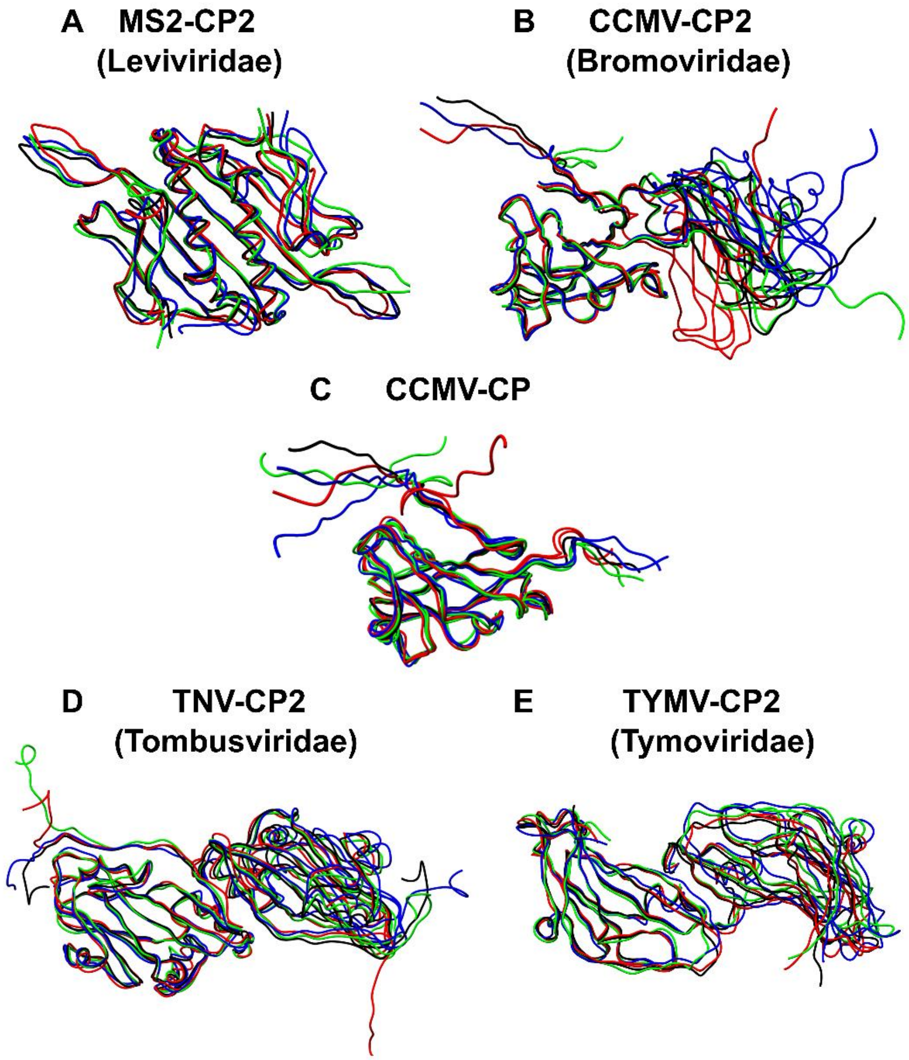

Figure S4. Flexibility of the dimerization interface. The left monomers of four CP2 structures from each representative virus were structurally aligned: the native structure (black), a structure whose COM distance is the mean of the COM distance distribution (green) and two structures whose COM distances are the mean-2×σ (red) and mean+2×σ (blue), where σ is the standard deviation of the COM distance distribution. The latter three structures were chosen at random from the simulations such that they satisfied the COM distance constraints. (A) Although only the left monomers of the bacteriophage MS2-CP2 were aligned, the right monomers also align well because the dimerization interface is not flexible. (B) The right monomer of the cowpea chlorotic mottle virus (CCMV) CP2 dimer is very mobile. (C) To show that the right monomer of CCMV does not unfold we aligned all the monomers (both left and right) in (B). It is clear that the monomers stay folded and only the termini are floppy. Thus, the motion in (B) is not due to right monomer unfolding but due to the flexibility of the dimerization interface. As expected, the right monomers of the (D) Tobacco necrosis virus (TNV) CP2 and the (E) Turnip yellow mosaic virus (TYMV) CP2 show intermediate levels of motion. Structure alignment was performed using the MatchMaker (Meng et al., 2006) option in UCSF-Chimera (Pettersen et al., 2004).

SUPPLEMENTARY TABLES

Table S1. List of *Leviviridae* capsids and the interface parameters derived from the tri-dimer structure. Interface: 2: dimerization, 3: trimerization, 5: pentamerization, 6: hexamerization. Chains: The chains between which the interface occurs. R and H are defined in the methods section. Nonpolar residues are defined in the SI Methods.

PDB-ID	Virus Name	Interface	Chains	R	H	Number of interface residues	Number of non-polar interface residues	% of interface non-polar residues
1DWN	PP7	2	AB	0.972	0.405	444	254	57.21
		2	BA	0.972	0.405	450	259	57.56
		2	CC	0.954	0.403	440	249	56.59
		3	AB	0.148	0.310	68	26	38.24
		3	AC	0.114	0.324	52	23	44.23
		3	BC	0.174	0.330	80	31	38.75
		5	BB	0.091	0.382	42	18	42.86
		6	AC	0.117	0.381	54	23	42.59
		6	CA	0.117	0.349	54	21	38.89
1FRS	FR	2	AB	1.045	0.417	506	311	61.46
		2	BA	1.077	0.418	516	316	61.24
		2	CC	0.983	0.428	466	290	62.23
		3	AB	0.141	0.333	68	36	52.94
		3	AC	0.143	0.314	68	31	45.59
		3	BC	0.150	0.283	72	25	34.72
		5	BB	0.131	0.380	64	35	54.69
		6	AC	0.143	0.377	68	39	57.35
		6	CA	0.147	0.369	70	39	55.71
1GAV	GA	2	AB	1.121	0.435	530	339	63.96
		2	BA	1.131	0.436	534	342	64.04
		2	CC	1.067	0.436	510	334	65.49
		3	AB	0.143	0.337	68	32	47.06
		3	AC	0.109	0.325	52	25	48.08
		3	BC	0.122	0.309	58	24	41.38
		5	BB	0.147	0.376	70	39	55.71
		6	AC	0.151	0.373	72	40	55.56
		6	CA	0.149	0.366	70	37	52.86
1QBE	Qβ	2	AB	1.169	0.418	526	335	63.69
		2	BA	1.173	0.419	528	336	63.64
		2	CC	1.198	0.420	526	345	65.59
		3	AB	0.156	0.273	70	25	35.71

		3	AC	0.142	0.256	62	24	38.71
		3	BC	0.181	0.263	82	31	37.80
		5	BB	0.107	0.295	50	19	38.00
		6	AC	0.106	0.265	46	12	26.09
		6	CA	0.110	0.233	48	12	25.00
2MS2	MS2	2	AB	1.090	0.424	510	315	61.76
		2	BA	1.090	0.425	510	316	61.96
		2	CC	1.043	0.436	482	304	63.07
		3	AB	0.155	0.341	72	37	51.39
		3	AC	0.147	0.332	68	31	45.59
		3	BC	0.159	0.311	74	29	39.19
		5	BB	0.145	0.369	68	36	52.94
		6	AC	0.147	0.379	68	39	57.35
		6	CA	0.151	0.364	70	37	52.86
2VF9	PRR1	2	AB	1.135	0.403	522	319	61.11
		2	BA	1.176	0.407	534	332	62.17
		2	CC	1.137	0.411	524	326	62.21
		3	AB	0.136	0.262	62	17	27.42
		3	AC	0.118	0.287	54	19	35.19
		3	BC	0.158	0.272	72	23	31.94
		5	BB	0.162	0.381	74	34	45.95
		6	AC	0.109	0.399	50	26	52.00
		6	CA	0.170	0.382	78	40	51.28
2W4Z	CB5	2	AB	1.145	0.389	490	295	60.20
		2	BA	1.145	0.390	490	297	60.61
		2	CC	1.084	0.389	476	287	60.29
		3	AB	0.126	0.261	54	22	40.74
		3	AC	0.106	0.265	46	21	45.65
		3	BC	0.079	0.225	34	11	32.35
		5	BB	0.075	0.377	32	17	53.12
		6	AC	0.060	0.353	26	13	50.00
		6	CA	0.060	0.398	26	15	57.69
5LQP	AP205	2	AB	1.424	0.346	588	286	48.64
		2	BA	1.442	0.347	594	296	49.83
		2	CC	1.489	0.356	594	294	49.49
		3	AB	0.083	0.360	34	14	41.18
		3	AC	0.059	0.350	24	9	37.50
		3	BC	0.098	0.322	40	14	35.00
		5	BB	0.107	0.419	44	25	56.82
		6	AC	0.104	0.385	42	18	42.86
		6	CA	0.113	0.381	46	23	50.00

Table S2. List of *Bromoviridae* capsids and the interface parameters derived from the tri-dimer structure. Interface: 2: dimerization, 3: trimerization, 5: pentamerization, 6: hexamerization. Chains: The chains between which the interface occurs. R and H are defined in the methods section. Nonpolar residues are defined in the SI Methods.

PDB-ID	Virus Name	Interface	Chains	R	H	Number of interface residues	Number of non-polar interface residues	% of interface non-polar residues
1CWP	CCMV	2	AB	0.279	0.333	206	106	51.46
		2	BA	0.281	0.332	206	105	50.97
		2	CC	0.278	0.350	208	116	55.77
		3	AB	0.112	0.259	82	31	37.80
		3	AC	0.084	0.258	62	23	37.10
		3	BC	0.095	0.262	70	27	38.57
		5	AA	0.111	0.304	82	37	45.12
		6	BC	0.211	0.416	156	91	58.33
		6	CB	0.209	0.415	156	92	58.97
1F15	CMV	2	AB	0.159	0.352	134	59	44.03
		2	BA	0.146	0.358	122	54	44.26
		2	CC	0.184	0.366	160	74	46.25
		3	AB	0.045	0.164	38	12	31.58
		3	AC	0.047	0.166	40	12	30.00
		3	BC	0.043	0.169	38	13	34.21
		5	AA	0.117	0.351	94	57	60.64
		6	BC	0.216	0.369	186	97	52.15
		6	CB	0.212	0.374	186	99	53.23
1JS9	BMV	2	AB	0.294	0.353	212	120	56.60
		2	BA	0.305	0.356	220	125	56.82
		2	CC	0.361	0.347	252	146	57.94
		3	AB	0.141	0.234	102	34	33.33
		3	AC	0.119	0.228	84	27	32.14
		3	BC	0.095	0.223	68	22	32.35
		5	AA	0.129	0.279	92	37	40.22
		6	BC	0.229	0.373	164	90	54.88
		6	CB	0.228	0.382	162	93	57.41
1LAJ	TAV	2	AB	0.184	0.322	148	64	43.24
		2	BA	0.154	0.319	124	53	42.74
		2	CC	0.179	0.338	150	68	45.33
		3	AB	0.060	0.244	48	16	33.33
		3	AC	0.057	0.246	46	15	32.61
		3	BC	0.048	0.266	40	15	37.50

		5	AA	0.183	0.351	142	58	40.85
		6	BC	0.218	0.345	180	73	40.56
		6	CB	0.201	0.348	168	68	40.48

Table S3. List of *Tombusviridae* capsids and the interface parameters derived from the tri-dimer structure. Interface: 2: dimerization, 3: trimerization, 5: pentamerization, 6: hexamerization. Chains: The chains between which the interface occurs. R and H are defined in the methods section. Nonpolar residues are defined in the SI Methods.

PDB-ID	Virus Name	Interface	Chains	R	H	Number of interface residues	Number of non-polar interface residues	% of interface non-polar residues
1C8N	TNV	2	AB	0.079	0.383	84	38	45.24
		2	BA	0.078	0.379	82	38	46.34
		2	CC	0.111	0.348	132	71	53.79
		3	AB	0.137	0.257	144	49	34.03
		3	AC	0.130	0.256	146	51	34.93
		3	BC	0.129	0.256	144	49	34.03
		5	AA	0.168	0.295	178	69	38.76
		6	BC	0.124	0.354	140	66	47.14
		6	CB	0.215	0.323	242	106	43.80
1F2N	CarMV	2	AB	0.086	0.295	90	34	37.78
		2	BA	0.081	0.298	86	33	38.37
		2	CC	0.260	0.347	276	113	40.94
		3	AB	0.137	0.279	144	45	31.25
		3	AC	0.127	0.265	134	37	27.61
		3	BC	0.200	0.304	210	77	36.67
		5	AA	0.131	0.320	140	59	42.14
		6	BC	0.078	0.272	82	28	34.15
		6	CB	0.145	0.321	152	65	42.76
1NG0	CFMV	2	AB	0.080	0.341	84	40	47.62
		2	BA	0.086	0.332	90	42	46.67
		2	CC	0.252	0.358	274	132	48.18
		3	AB	0.134	0.287	140	42	30.00
		3	AC	0.121	0.272	128	36	28.12
		3	BC	0.193	0.290	206	71	34.47
		5	AA	0.157	0.253	164	49	29.88
		6	BC	0.089	0.238	96	26	27.08
		6	CB	0.148	0.258	158	45	28.48
1OPO	RYMV	2	AB	0.138	0.329	216	106	49.07
		2	BA	0.135	0.333	210	101	48.10
		2	CC	0.119	0.347	186	92	49.46
		3	AB	0.055	0.204	86	17	19.77
		3	AC	0.056	0.200	88	17	19.32
		3	BC	0.058	0.193	90	17	18.89

		5	AA	0.097	0.291	152	60	39.47
		6	BC	0.094	0.296	148	61	41.22
		6	CB	0.097	0.304	152	62	40.79
1SMV	SMV	2	AB	0.063	0.460	70	47	67.14
		2	BA	0.061	0.460	68	47	69.12
		2	CC	0.091	0.365	112	53	47.32
		3	AB	0.140	0.295	154	60	38.96
		3	AC	0.137	0.288	160	61	38.12
		3	BC	0.123	0.283	144	53	36.81
		5	AA	0.148	0.300	164	49	29.88
		6	BC	0.159	0.294	186	72	38.71
		6	CB	0.152	0.305	178	58	32.58
2IZW	RMV	2	AB	0.143	0.262	140	62	44.29
		2	BA	0.139	0.265	136	62	45.59
		2	CC	0.088	0.292	94	35	37.23
		3	AB	0.164	0.332	160	62	38.75
		3	AC	0.155	0.333	158	61	38.61
		3	BC	0.168	0.326	170	64	37.65
		5	AA	0.130	0.274	128	46	35.94
		6	BC	0.186	0.279	190	74	38.95
		6	CB	0.154	0.276	156	56	35.90
2TBV	TBSV	2	AB	0.214	0.410	366	205	56.01
		2	BA	0.215	0.412	368	207	56.25
		2	CC	0.231	0.375	414	220	53.14
		3	AB	0.067	0.255	114	37	32.46
		3	AC	0.067	0.259	116	38	32.76
		3	BC	0.061	0.239	106	30	28.30
		5	AA	0.087	0.309	148	71	47.97
		6	BC	0.122	0.312	214	101	47.20
		6	CB	0.089	0.313	156	70	44.87
2ZAH	MNSV	2	AB	0.181	0.371	322	183	56.83
		2	BA	0.179	0.374	320	181	56.56
		2	CC	0.193	0.333	368	182	49.46
		3	AB	0.056	0.222	100	31	31.00
		3	AC	0.056	0.225	102	30	29.41
		3	BC	0.056	0.219	104	32	30.77
		5	AA	0.090	0.280	158	52	32.91
		6	BC	0.115	0.296	214	80	37.38
		6	CB	0.097	0.308	180	66	36.67
4SBV	SBMV	2	AB	0.087	0.377	96	51	53.12
		2	BA	0.089	0.379	98	52	53.06
		2	CC	0.096	0.328	118	61	51.69
		3	AB	0.155	0.329	170	63	37.06

		3	AC	0.143	0.324	166	61	36.75
		3	BC	0.146	0.324	170	60	35.29
		5	AA	0.158	0.294	174	54	31.03
		6	BC	0.141	0.296	164	62	37.80
		6	CB	0.160	0.278	186	56	30.11
6MRL	CLSV	2	AB	0.205	0.369	364	189	51.92
		2	BA	0.200	0.369	354	183	51.69
		2	CC	0.149	0.325	280	125	44.64
		3	AB	0.057	0.219	102	27	26.47
		3	AC	0.059	0.226	108	28	25.93
		3	BC	0.060	0.205	110	23	20.91
		5	AA	0.084	0.292	148	55	37.16
		6	BC	0.140	0.298	256	95	37.11
		6	CB	0.093	0.313	170	70	41.18
6MRM	RCNMV	2	AB	0.198	0.359	350	158	45.14
		2	BA	0.199	0.358	352	159	45.17
		2	CC	0.167	0.312	310	124	40.00
		3	AB	0.058	0.240	102	30	29.41
		3	AC	0.054	0.249	98	29	29.59
		3	BC	0.059	0.242	106	29	27.36
		5	AA	0.087	0.281	154	50	32.47
		6	BC	0.145	0.297	262	89	33.97
		6	CB	0.082	0.304	148	53	35.81

Table S4. List of *Tymoviridae* capsids and the interface parameters derived from the tri-dimer structure. Interface: 2: dimerization, 3: trimerization, 5: pentamerization, 6: hexamerization. Chains: The chains between which the interface occurs. R and H are defined in the methods section. Nonpolar residues are defined in the SI Methods.

PDB-ID	Virus Name	Interface	Chains	R	H	Number of interface residues	Number of non-polar interface residues	% of interface non-polar residues
1AUY	TYMV	2	AB	0.138	0.354	126	47	37.30
		2	BA	0.137	0.356	124	45	36.29
		2	CC	0.136	0.349	130	47	36.15
		3	AB	0.119	0.356	108	43	39.81
		3	AC	0.149	0.361	134	50	37.31
		3	BC	0.196	0.408	188	89	47.34
		5	AA	0.163	0.342	138	56	40.58
		6	BC	0.188	0.337	182	69	37.91
		6	CB	0.203	0.319	196	72	36.73
1DDL	DYMV	2	AB	0.160	0.423	134	76	56.72
		2	BA	0.162	0.432	136	80	58.82
		2	CC	0.138	0.436	114	72	63.16
		3	AB	0.353	0.298	294	97	32.99
		3	AC	0.235	0.293	196	58	29.59
		3	BC	0.335	0.293	276	88	31.88
		5	AA	0.165	0.293	140	58	41.43
		6	BC	0.145	0.299	120	53	44.17
		6	CB	0.145	0.293	120	49	40.83
1E57	PMV	2	AB	0.130	0.429	116	63	54.31
		2	BA	0.130	0.429	116	63	54.31
		2	CC	0.122	0.441	116	64	55.17
		3	AB	0.094	0.359	84	43	51.19
		3	AC	0.128	0.368	114	55	48.25
		3	BC	0.175	0.361	166	71	42.77
		5	AA	0.183	0.370	154	84	54.55
		6	BC	0.122	0.404	116	59	50.86
		6	CB	0.150	0.373	142	75	52.82

SUPPLEMENTARY DATA

Data S1. Multiple Sequence Alignment (MSA) of A, B and C chains from the IAUs of *Leviviridae* structures. The MSAs in Data S1, S2, S3 and S4 were performed using Clustal-W (Thompson et al., 2003) on the T-Coffee webserver (di Tommaso et al., 2011). All conformers (A, B and C) are shown here despite having the same sequence in order to underline that some chains have missing residues and these residues were not rebuilt for calculating contact maps. Some dispersion in the values of H and R may occur because of such missing residues.

```
1DWN_A_127     1 --SKTIVLSVGEATRTLTEIQSTADRQIF--------EE   29
1DWN_B_127     1 --SKTIVLSVGEATRTLTEIQSTADRQIF--------EE   29
1DWN_C_127     1 --SKTIVLSVGEATRTLTEIQSTADRQIF--------EE   29
1FRS_A_129     1 -ASNFEEFVLVDNGGTGDVKVAPSNFANG-----VAEWI   33
1FRS_B_129     1 -ASNFEEFVLVDNGGTGDVKVAPSNFANG-----VAEWI   33
1FRS_C_129     1 -ASNFEEFVLVDNGGTGDVKVAPSNFANG-----VAEWI   33
1GAV_A_129     1 --ATLRSFVLVDNGGTGNVTVVPVSNANG-----VAEWL   32
1GAV_B_129     1 --ATLRSFVLVDNGGTGNVTVVPVSNANG-----VAEWL   32
1GAV_C_129     1 --ATLRSFVLVDNGGTGNVTVVPVSNANG-----VAEWL   32
1QBE_A_123     1 --AKLETVTLGNIGKDGKQTLVLNPRGVNPTNGVASLSQ   37
1QBE_B_132     1 --AKLETVTLGNIGKDGKQTLVLNPRGVNPTNGVASLSQ   37
1QBE_C_123     1 --AKLETVTLGNIGKDGKQTLVLNPRGVNPTNGVASLSQ   37
2MS2_A_129     1 -ASNFTQFVLVDNGGTGDVTVAPSNFANG-----VAEWI   33
2MS2_B_129     1 -ASNFTQFVLVDNGGTGDVTVAPSNFANG-----VAEWI   33
2MS2_C_129     1 -ASNFTQFVLVDNGGTGDVTVAPSNFANG-----VAEWI   33
2VF9_A_131     1 --AQLQNLVLKDR-EATPNDHTFVPRDIR--DNVGEVVE   34
2VF9_B_131     1 --AQLQNLVLKDR-EATPNDHTFVPRDIR--DNVGEVVE   34
2VF9_C_131     1 --AQLQNLVLKDR-EATPNDHTFVPRDIR--DNVGEVVE   34
2W4Z_A_122     1 ALGDTLTITLGGSGGTAKVLRKINQDGYT---------S   30
2W4Z_B_122     1 ALGDTLTITLGGSGGTAKVLRKINQDGYT---------S   30
2W4Z_C_122     1 ALGDTLTITLGGSGGTAKVLRKINQDGYT---------S   30
5LQP_A_129     1 -----ANKPMQPITSTANKIVWSDPTRLS---------T   25
5LQP_B_129     1 -----ANKPMQPITSTANKIVWSDPTRLS---------T   25
5LQP_C_129     1 -----ANKPMQPITSTANKIVWSDPTRLS---------T   25
cons           1          :                              39
```

```
1DWN_A_127       30 KVGPLVGRLRLTASLRQNGAKT-AYRVNLKLDQADVVDC   67
1DWN_B_127       30 KVGPLVGRLRLTASLRQNGAKT-AYRVNLKLDQADVVDC   67
1DWN_C_127       30 KVGPLVGRLRLTASLRQNGAKT-AYRVNLKLDQADVVDC   67
1FRS_A_129       34 SSNSRSQAYKVTCSVRQSSAN--NRKYTVKVEVPKVATQ   70
1FRS_B_129       34 SSNSRSQAYKVTCSVRQSSAN--NRKYTVKVEVPKVATQ   70
1FRS_C_129       34 SSNSRSQAYKVTCSVRQSSAN--NRKYTVKVEVPKVATQ   70
1GAV_A_129       33 SNNSRSQAYRVTASYRASGAD--KRKYTIKLEVPKIVTQ   69
1GAV_B_129       33 SNNSRSQAYRVTASYRASGAD--KRKYTIKLEVPKIVTQ   69
1GAV_C_129       33 SNNSRSQAYRVTASYRASGAD--KRKYTIKLEVPKIVTQ   69
1QBE_A_123       38 AGAVPALEKRVTVSVSQP-----NYKVQVKIQNPTACT-   70
1QBE_B_132       38 AGAVPALEKRVTVSVSQPSRNRKNYKVQVKIQNPTACTA   76
1QBE_C_123       38 AGAVPALEKRVTVSVSQP-----NYKVQVKIQNPTACT-   70
2MS2_A_129       34 SSNSRSQAYKVTCSVRQSSAQ--NRKYTIKVEVPKVATQ   70
2MS2_B_129       34 SSNSRSQAYKVTCSVRQSSAQ--NRKYTIKVEVPKVATQ   70
2MS2_C_129       34 SSNSRSQAYKVTCSVRQSSAQ--NRKYTIKVEVPKVATQ   70
2VF9_A_131       35 STGVPIGESRFTISLRKTSNG--RYKSTLKLVVPVVQSQ   71
2VF9_B_131       35 STGVPIGESRFTISLRKTSNG--RYKSTLKLVVPVVQSQ   71
2VF9_C_131       35 STGVPIGESRFTISLRKTSNG--RYKSTLKLVVPVVQSQ   71
2W4Z_A_122       31 EYYLPETSSSFRAKVRHTKESVKPNQVQYERHNVEFTET   69
2W4Z_B_122       31 EYYLPETSSSFRAKVRHTKESVKPNQVQYERHNVEFTET   69
2W4Z_C_122       31 EYYLPETSSSFRAKVRHTKESVKPNQVQYERHNVEFTET   69
5LQP_A_129       26 TFSASLLRQRVKVGIAELNNVSGQYVSVYKRPAPKPEGC   64
5LQP_B_129       26 TFSASLLRQRVKVGIAELNNVSGQYVSVYKRPAPKPEGC   64
5LQP_C_129       26 TFSASLLRQRVKVGIAELNNVSGQYVSVYKRPAPKPEGC   64
cons             40           .                  :            78
```

```
1DWN_A_127    68 STSVCGELPKVRYTQVWSHDVTIVANSTEASRKSLYDLT 106
1DWN_B_127    68 STSVCGELPKVRYTQVWSHDVTIVANSTEASRKSLYDLT 106
1DWN_C_127    68 STSVCGELPKVRYTQVWSHDVTIVANSTEASRKSLYDLT 106
1FRS_A_129    71 VQGG-VELPVAAWRSYMNMELTIPVFATNDDCALIVKAL 108
1FRS_B_129    71 VQGG-VELPVAAWRSYMNMELTIPVFATNDDCALIVKAL 108
1FRS_C_129    71 VQGG-VELPVAAWRSYMNMELTIPVFATNDDCALIVKAL 108
1GAV_A_129    70 VVNG-VELPVSAWKAYASIDLTIPIFAATDDVTVISKSL 107
1GAV_B_129    70 VVNG-VELPVSAWKAYASIDLTIPIFAATDDVTVISKSL 107
1GAV_C_129    70 VVNG-VELPVSAWKAYASIDLTIPIFAATDDVTVISKSL 107
1QBE_A_123    71 ------CDPSVTRQAYADVTFSFTQYSTDEER-AFVRTE 102
1QBE_B_132    77 NGS---CDPSVTRQAYADVTFSFTQYSTDEER-AFVRTE 111
1QBE_C_123    71 ------CDPSVTRQAYADVTFSFTQYSTDEER-AFVRTE 102
2MS2_A_129    71 TVGG-VELPVAAWRSYLNMELTIPIFATNSDCELIVKAM 108
2MS2_B_129    71 TVGG-VELPVAAWRSYLNMELTIPIFATNSDCELIVKAM 108
2MS2_C_129    71 TVGG-VELPVAAWRSYLNMELTIPIFATNSDCELIVKAM 108
2VF9_A_131    72 TVNGI-VTPVVVRTSYVTVDFDYDARSTTKERNNFVGMI 109
2VF9_B_131    72 TVNGI-VTPVVVRTSYVTVDFDYDARSTTKERNNFVGMI 109
2VF9_C_131    72 TVNGI-VTPVVVRTSYVTVDFDYDARSTTKERNNFVGMI 109
2W4Z_A_122    70 VYAS-GSTPEFVRQAYVVIRHKVGDVSA-----TVSDLG 102
2W4Z_B_122    70 VYAS-GSTPEFVRQAYVVIRHKVGDVSA-----TVSDLG 102
2W4Z_C_122    70 VYAS-GSTPEFVRQAYVVIRHKVGDVSA-----TVSDLG 102
5LQP_A_129    65 ADAC-VIMPNENQSIRTVISGSAENLATLKAEWETHKRN 102
5LQP_B_129    65 ADAC-VIMPNENQSIRTVISGSAENLATLKAEWETHKRN 102
5LQP_C_129    65 ADAC-VIMPNENQSIRTVISGSAENLATLKAEWETHKRN 102
cons          79         *                 ::            117
```

```
1DWN_A_127      107 KSLVATS--QVEDLVVNLVPLGR----  127
1DWN_B_127      107 KSLVATS--QVEDLVVNLVPLGR----  127
1DWN_C_127      107 KSLVATS--QVEDLVVNLVPLGR----  127
1FRS_A_129      109 QGTFKTGN-PIATAIAANSGIY-----  129
1FRS_B_129      109 QGTFKTGN-PIATAIAANSGIY-----  129
1FRS_C_129      109 QGTFKTGN-PIATAIAANSGIY-----  129
1GAV_A_129      108 AGLFKVGN-PIAEAISSQSGFYA----  129
1GAV_B_129      108 AGLFKVGN-PIAEAISSQSGFYA----  129
1GAV_C_129      108 AGLFKVGN-PIAEAISSQSGFYA----  129
1QBE_A_123      103 LAALLASPLLI-DAIDQLNPAY-----  123
1QBE_B_132      112 LAALLASPLLI-DAIDQLNPAY-----  132
1QBE_C_123      103 LAALLASPLLI-DAIDQLNPAY-----  123
2MS2_A_129      109 QGLLKDGN-PIPSAIAANSGIY-----  129
2MS2_B_129      109 QGLLKDGN-PIPSAIAANSGIY-----  129
2MS2_C_129      109 QGLLKDGN-PIPSAIAANSGIY-----  129
2VF9_A_131      110 ADALKADKMLVHDTIVNLQGVY-----  131
2VF9_B_131      110 ADALKADKMLVHDTIVNLQGVY-----  131
2VF9_C_131      110 ADALKADKMLVHDTIVNLQGVY-----  131
2W4Z_A_122      103 EALSFYLNEALYGKLIGWES-------  122
2W4Z_B_122      103 EALSFYLNEALYGKLIGWES-------  122
2W4Z_C_122      103 EALSFYLNEALYGKLIGWES-------  122
5LQP_A_129      103 VDTLFASGNAGLGFLDPTAAIVSSDTT  129
5LQP_B_129      103 VDTLFASGNAGLGFLDPTAAIVSSDTT  129
5LQP_C_129      103 VDTLFASGNAGLGFLDPTAAIVSSDTT  129
cons            118               :              144
```

BAD AVG GOOD

Data S2: MSA of A, B and C chains from the IAUs of all *Bromoviridae* structures.

```
1CWP_A_149    1 ---------------------------------------    0
1CWP_B_164    1 -------------------------VVQPVIVEPIASGQ   14
1CWP_C_164    1 -------------------------VVQPVIVEPIASGQ   14
1F15_A_157    1 ---------------------------------------    0
1F15_B_190    1 ---------DANFRVLSQQLSRLNKTLAAGRPTINHPTF   30
1F15_C_191    1 --------ADANFRVLSQQLSRLNKTLAAGRPTINHPTF   31
1JS9_A_149    1 ---------------------------------------    0
1JS9_B_165    1 ------------------------ARVQPVIVEPLAAGQ   15
1JS9_C_189    1 MSTSGTGKMTRAQRRAAARRNRWTARVQPVIVEPLAAGQ   39
1LAJ_A_174    1 ------------------------NIASSSAPSLQHPTF   15
1LAJ_B_182    1 ----------------TQQVNRLANIASSSAPSLQHPTF   23
1LAJ_C_184    1 --------------ALTQQVNRLANIASSSAPSLQHPTF   25
cons          1                                           39

1CWP_A_149    1 -KAIKAWTGYSVSKWTASCAAAEAKVTSAITISLPNELS   38
1CWP_B_164   15 GKAIKAWTGYSVSKWTASCAAAEAKVTSAITISLPNELS   53
1CWP_C_164   15 GKAIKAWTGYSVSKWTASCAAAEAKVTSAITISLPNELS   53
1F15_A_157    1 ---ERCRPGYTFTSITLKPPKIDRGSYYGKRLLLPDSVT   36
1F15_B_190   31 VGSERCRPGYTFTSITLKPPKIDRGSYYGKRLLLPDSVT   69
1F15_C_191   32 VGSERCRPGYTFTSITLKPPKIDRGSYYGKRLLLPDSVT   70
1JS9_A_149    1 -KAIKAIAGYSISKWEASSDAITAKATNAMSITLPHELS   38
1JS9_B_165   16 GKAIKAIAGYSISKWEASSDAITAKATNAMSITLPHELS   54
1JS9_C_189   40 GKAIKAIAGYSISKWEASSDAITAKATNAMSITLPHELS   78
1LAJ_A_174   16 IASKKCRAGYTYTSLDVRPTRTEKDKSFGQRLIIPVPVS   54
1LAJ_B_182   24 IASKKCRAGYTYTSLDVRPTRTEKDKSFGQRLIIPVPVS   62
1LAJ_C_184   26 IASKKCRAGYTYTSLDVRPTRTEKDKSFGQRLIIPVPVS   64
cons         40     :. .**: :.              .  : :*  ::   78

1CWP_A_149   39 SERNK-----QLKVG-------RVLLWLGLLPSVSGTVK   65
1CWP_B_164   54 SERNK-----QLKVG-------RVLLWLGLLPSVSGTVK   80
1CWP_C_164   54 SERNK-----QLKVG-------RVLLWLGLLPSVSGTVK   80
1F15_A_157   37 EYDKKLVSRLQIRVNPLPKFDSTVWVTVRKVPASSDLSV   75
1F15_B_190   70 EYDKKLVSRLQIRVNPLPKFDSTVWVTVRKVPASSDLSV  108
1F15_C_191   71 EYDKKLVSRLQIRVNPLPKFDSTVWVTVRKVPASSDLSV  109
1JS9_A_149   39 SEKNK-----ELKVG-------RVLLWLGLLPSVAGRIK   65
1JS9_B_165   55 SEKNK-----ELKVG-------RVLLWLGLLPSVAGRIK   81
1JS9_C_189   79 SEKNK-----ELKVG-------RVLLWLGLLPSVAGRIK  105
1LAJ_A_174   55 EYPKKKVSCVQVRLNPSPKFNSTIWVSLRRLDETTLLTS   93
1LAJ_B_182   63 EYPKKKVSCVQVRLNPSPKFNSTIWVSLRRLDETTLLTS  101
1LAJ_C_184   65 EYPKKKVSCVQVRLNPSPKFNSTIWVSLRRLDETTLLTS  103
cons         79 .  :*     ::::.        : : :  :   :      117
```

```
1CWP_A_149        66 SCVTETQTTAAASFQVALAVADNSKDVVAAMYPEAFKGI  104
1CWP_B_164        81 SCVTETQTTAAASFQVALAVADNSKDVVAAMYPEAFKGI  119
1CWP_C_164        81 SCVTETQTTAAASFQVALAVADNSKDVVAAMYPEAFKGI  119
1F15_A_157        76 AAISAMFADGASPVLVYQYAASGVQANNKLLYDLSAMRA  114
1F15_B_190       109 AAISAMFADGASPVLVYQYAASGVQANNKLLYDLSAMRA  147
1F15_C_191       110 AAISAMFADGASPVLVYQYAASGVQANNKLLYDLSAMRA  148
1JS9_A_149        66 ACVAEKQAQAEAAFQVALAVADSSKEVVAAMYTDAFRGA  104
1JS9_B_165        82 ACVAEKQAQAEAAFQVALAVADSSKEVVAAMYTDAFRGA  120
1JS9_C_189       106 ACVAEKQAQAEAAFQVALAVADSSKEVVAAMYTDAFRGA  144
1LAJ_A_174        94 ENVFKLFTDGLA-VLIYQHVPTGIQPNNKITFDMSNVGA  131
1LAJ_B_182       102 ENVFKLFTDGLA-VLIYQHVPTGIQPNNKITFDMSNVGA  139
1LAJ_C_184       104 ENVFKLFTDGLA-VLIYQHVPTGIQPNNKITFDMSNVGA  141
cons             118   :    : . : . :   .. . :      :  :      156

1CWP_A_149       105 TLEQLAADLTIYLYSSAALTEGDVIVHLEVEHVRPTFDD  143
1CWP_B_164       120 TLEQLAADLTIYLYSSAALTEGDVIVHLEVEHVRPTFDD  158
1CWP_C_164       120 TLEQLAADLTIYLYSSAALTEGDVIVHLEVEHVRPTFDD  158
1F15_A_157       115 DIGDMRKYAVLVYSKDDALETDELVLHVDIEHQRIPTSG  153
1F15_B_190       148 DIGDMRKYAVLVYSKDDALETDELVLHVDIEHQRIPTSG  186
1F15_C_191       149 DIGDMRKYAVLVYSKDDALETDELVLHVDIEHQRIPTSG  187
1JS9_A_149       105 TLGDLLN-LQIYLYASEAVPAKAVVVHLEVEHVRPTFDD  142
1JS9_B_165       121 TLGDLLN-LQIYLYASEAVPAKAVVVHLEVEHVRPTFDD  158
1JS9_C_189       145 TLGDLLN-LQIYLYASEAVPAKAVVVHLEVEHVRPTFDD  182
1LAJ_A_174       132 EIGDMGKYALIVYSKDDVLEADEMVIHIDIEHQRIPSAS  170
1LAJ_B_182       140 EIGDMGKYALIVYSKDDVLEADEMVIHIDIEHQRIPSAS  178
1LAJ_C_184       142 EIGDMGKYALIVYSKDDVLEADEMVIHIDIEHQRIPSAS  180
cons             157  : ::     :    . .:    :::*:::** * .  .  195

1CWP_A_149       144 SFTPVY-  149
1CWP_B_164       159 SFTPVY-  164
1CWP_C_164       159 SFTPVY-  164
1F15_A_157       154 VLPV---  157
1F15_B_190       187 VLPV---  190
1F15_C_191       188 VLPV---  191
1JS9_A_149       143 FFTPVYR  149
1JS9_B_165       159 FFTPVYR  165
1JS9_C_189       183 FFTPVYR  189
1LAJ_A_174       171 TLPV---  174
1LAJ_B_182       179 TLPV---  182
1LAJ_C_184       181 TLPV---  184
cons             196  :.      202
```

BAD AVG GOOD

Data S3: MSA of A, B and C chains from the IAUs of all *Tombusviridae* structures.

```
1C8N_A_189      1 -----------------------------------NSTV   4
1C8N_B_189      1 ------------------------------------STV   3
1C8N_C_219      1 -----GVSRAGGFVTAPVIGAMVTRPTVPRFGMRGNSTV  34
1F2N_A_189      1 --------------------------------LSSNTWP   7
1F2N_B_189      1 --------------------------------LSSNTWP   7
1F2N_C_212      1 ---------AEPQLQRAPVAQASRISGTVPGPLSSNTWP  30
1NG0_A_192      1 ----------------------------------DWFDT   5
1NG0_B_192      1 ----------------------------------DWFDT   5
1NG0_C_218      1 --------VSRPLNPPAAVGSTLKAGRGRTAGVSDWFDT  31
1OPO_A_267      1 ------------------------------------SMT   3
1OPO_B_267      1 ------------------------------------SMT   3
1OPO_C_268      1 -----------------------------------KSMT   4
1SMV_A_196      1 -----------------------------------GAIT   4
1SMV_B_196      1 -----------------------------------GAIT   4
1SMV_C_222      1 ---------QAGISMAPSAQGAMVRIRNPAVSSSRGAIT  30
2IZW_A_178      1 --------------------------------QYGDITP   7
2IZW_B_179      1 -------------------------------TQYGDITP   8
2IZW_C_208      1 --GQQPTRQVTPVSAPAAMGTQITYRGPQVVTQYGDITP  37
2TBV_A_287      1 -----------------------------------GVTV   4
2TBV_B_286      1 -----------------------------------GVTV   4
2TBV_C_322      1 IITHVGGVGGSIMAPVAVSRQLVGSKPKFTGRTSGGVTV  39
2ZAH_A_296      1 -----------------------------------SVKI   4
2ZAH_B_297      1 -----------------------------------SVKI   4
2ZAH_C_331      1 NISYTEGAKPGAISAPVAISRRVAGMKPRFVRSEGSVKI  39
4LLF_A_290      1 ----------------------------------GSVRI   5
4LLF_B_290      1 ----------------------------------GSVRI   5
4LLF_B_292      1 --------------------------------AKGSVRI   7
4LLF_C_323      1 IAHHPQAFPGAIAAPISYAYAVKGRKPRFQ-TAKGSVRI  38
4SBV_A_199      1 --------------------------------SSMDVTI   7
4SBV_B_199      1 --------------------------------SSMDVTI   7
4SBV_C_222      1 ---------QAGVSMAPIAQGTMVKLRPPMLRSSMDVTI  30
6MRL_A_295      1 ----------------------------------GKVHI   5
6MRL_B_295      1 ----------------------------------GKVHI   5
6MRL_C_326      1 ---NQIVGGIGAIAAPVSITKRVRGMRPSFRQTKGKVHI  36
6MRM_A_291      1 ------------------------------------RGI   3
6MRM_B_291      1 ------------------------------------RGI   3
6MRM_C_315      1 ------------TPNTSVKTVAIPFAKTQIIKTVNPPPI  27
cons            1                                          39
```

```
1C8N_A_189         5 VSNSELILNLTPIALAYTVQ------SLPL----I----    29
1C8N_B_189         4 VSNSELILNLTPIALAYTVQ------SLPL----I----    28
1C8N_C_219        35 VSNSELILNLTPIALAYTVQ------SLPL----I----    59
1F2N_A_189         8 LHSVEFLADFKRSSTSADAT------TYDC----VP---    33
1F2N_B_189         8 LHSVEFLADFKRSSTSADAT------TYDC----VP---    33
1F2N_C_212        31 LHSVEFLADFKRSSTSADAT------TYDC----VP---    56
1NG0_A_192         6 GMITSYLGGFQRTAGTTDSQ------VFIV----SP---    31
1NG0_B_192         6 GMITSYLGGFQRTAGTTDSQ------VFIV----SP---    31
1NG0_C_218        32 GMITSYLGGFQRTAGTTDSQ------VFIV----SP---    57
1OPO_A_267         4 MSKTELLSTVKGTTGVIPSF-------EDW----VVSPR    31
1OPO_B_267         4 MSKTELLSTVKGTTGVIPSF-------EDW----VVSPR    31
1OPO_C_268         5 MSKTELLSTVKGTTGVIPSF-------EDW----VVSPR    32
1SMV_A_196         5 VLHCELTAEIGVTDSIVVSS------ELVM----PY---    30
1SMV_B_196         5 VLHCELTAEIGVTDSIVVSS------ELVM----PY---    30
1SMV_C_222        31 VLHCELTAEIGVTDSIVVSS------ELVM----PY---    56
2IZW_A_178         8 AKNSGSLVRVTSSATAGTEV------SGTV----LFNVR    36
2IZW_B_179         9 AKNSGSLVRVTSSATAGTEV------SGTV----LFNVR    37
2IZW_C_208        38 AKNSGSLVRVTSSATAGTEV------SGTV----LFNVR    66
2TBV_A_287         5 TSHREYLTQVNNSSGFVVNG-GIVG--NSL----QLNPS    36
2TBV_B_286         5 TSHREYLTQVNNSSGFVVNG-GIVG--NSL----QLNPS    36
2TBV_C_322        40 TSHREYLTQVNNSSGFVVNG-GIVG--NSL----QLNPS    71
2ZAH_A_296         5 VHREFIASVLPSN-DLTVNN-GDVNIGKYR-----VNPS    36
2ZAH_B_297         5 VHREFIASVLPSN-DLTVNN-GDVNIGKYR----RVNPS    37
2ZAH_C_331        40 VHREFIASVLPSN-DLTVNN-GDVNIGKYR-----VNPS    71
4LLF_A_290         6 THRREYVSVLSGTNGEFLRN-NGTGPNNDF----SINPL    39
4LLF_B_290         6 THRREYVSVLSGTNGEFLRN-NGTGPNNDF----SINPL    39
4LLF_B_292         8 THRREYVSVLSGTNGEFLRN-NGTGPNNDF----SINPL    41
4LLF_C_323        39 THRREYVSVLSGTNGEFLRN-NGTGPNNDF----SINPL    72
4SBV_A_199         8 LSHCELSTELAVTVTIVVTS------ELVM----PF---    33
4SBV_B_199         8 LSHCELSTELAVTVTIVVTS------ELVM----PF---    33
4SBV_C_222        31 LSHCELSTELAVTVTIVVTS------ELVM----PF---    56
6MRL_A_295         6 VHRELVTSVINLVGNFRVNNNVSAQIGQFR-----INPS    39
6MRL_B_295         6 VHRELVTSVINLVGNFRVNNNVSAQIGQFR-----INPS    39
6MRL_C_326        37 VHRELVTSVINLVGNFRVNNNVSAQIGQFR-----INPS    70
6MRM_A_291         4 LHTQLVMSVVGSV-QMRTNN--GKSNQRFRLNPSN----    35
6MRM_B_291         4 LHTQLVMSVVGSV-QMRTNN--GKSNQRFRLNPSN----    35
6MRM_C_315        28 LHTQLVMSVVGSV-QMRTNN--GKSNQRFRLNPSN----    59
cons              40          .                                 78
```

```
1C8N_A_189        30 -ATQPAWLGTIADNYSKWRWVSLRIIYSPKCPTTTSGTV   67
1C8N_B_189        29 -ATQPAWLGTIADNYSKWRWVSLRIIYSPKCPTTTSGTV   66
1C8N_C_219        60 -ATQPAWLGTIADNYSKWRWVSLRIIYSPKCPTTTSGTV   97
1F2N_A_189        34 --FNLPRVWSLARCYSMWKPTRWDVVYLPEVSATVAGSI   70
1F2N_B_189        34 --FNLPRVWSLARCYSMWKPTRWDVVYLPEVSATVAGSI   70
1F2N_C_212        57 --FNLPRVWSLARCYSMWKPTRWDVVYLPEVSATVAGSI   93
1NG0_A_192        32 --AALDRVGTIAKAYALWRPKHWEIVYLPRCSTQTDGSI   68
1NG0_B_192        32 --AALDRVGTIAKAYALWRPKHWEIVYLPRCSTQTDGSI   68
1NG0_C_218        58 --AALDRVGTIAKAYALWRPKHWEIVYLPRCSTQTDGSI   94
1OPO_A_267        32 NVAVFPQLSLLATNFNKYRITALTVKYSPACSFETNGRV   70
1OPO_B_267        32 NVAVFPQLSLLATNFNKYRITALTVKYSPACSFETNGRV   70
1OPO_C_268        33 NVAVFPQLSLLATNFNKYRITALTVKYSPACSFETNGRV   71
1SMV_A_196        31 --TVGTWLRGVADNWSKYSWLSVRYTYIPSCPSSTAGSI   67
1SMV_B_196        31 --TVGTWLRGVADNWSKYSWLSVRYTYIPSCPSSTAGSI   67
1SMV_C_222        57 --TVGTWLRGVADNWSKYSWLSVRYTYIPSCPSSTAGSI   93
2IZW_A_178        37 NATELPWLSGQGSRYSKYRVRYAHFTWEPIVGSNTNGEV   75
2IZW_B_179        38 NATELPWLSGQGSRYSKYRVRYAHFTWEPIVGSNTNGEV   76
2IZW_C_208        67 NATELPWLSGQGSRYSKYRVRYAHFTWEPIVGSNTNGEV  105
2TBV_A_287        37 NGTLFSWLPALASNFDQYSFNSVVLDYVPLCGTTEVGRV   75
2TBV_B_286        37 NGTLFSWLPALASNFDQYSFNSVVLDYVPLCGTTEVGRV   75
2TBV_C_322        72 NGTLFSWLPALASNFDQYSFNSVVLDYVPLCGTTEVGRV  110
2ZAH_A_296        37 NNALFTWLQGQAQLYDMYRFTRLRFTYIPTTGSTSTGRV   75
2ZAH_B_297        38 NNALFTWLQGQAQLYDMYRFTRLRFTYIPTTGSTSTGRV   76
2ZAH_C_331        72 NNALFTWLQGQAQLYDMYRFTRLRFTYIPTTGSTSTGRV  110
4LLF_A_290        40 NPFLFPWLVNIAANFDQYKFNSLRFEYVPLVNTTTNGRV   78
4LLF_B_290        40 NPFLFPWLVNIAANFDQYKFNSLRFEYVPLVNTTTNGRV   78
4LLF_B_292        42 NPFLFPWLVNIAANFDQYKFNSLRFEYVPLVNTTTNGRV   80
4LLF_C_323        73 NPFLFPWLVNIAANFDQYKFNSLRFEYVPLVNTTTNGRV  111
4SBV_A_199        34 --TVGTWLRGVAQNWSKYAWVAIRYTYLPSCPTTTSGAI   70
4SBV_B_199        34 --TVGTWLRGVAQNWSKYAWVAIRYTYLPSCPTTTSGAI   70
4SBV_C_222        57 --TVGTWLRGVAQNWSKYAWVAIRYTYLPSCPTTTSGAI   93
6MRL_A_295        40 NSSLFTWLPTIASNFDSYRFTSIRFVYVPLCATTETGRV   78
6MRL_B_295        40 NSSLFTWLPTIASNFDSYRFTSIRFVYVPLCATTETGRV   78
6MRL_C_326        71 NSSLFTWLPTIASNFDSYRFTSIRFVYVPLCATTETGRV  109
6MRM_A_291        36 -PALFPTLAYEAANYDMYRLKKLTLRYVPLVTVQNSGRV   73
6MRM_B_291        36 -PALFPTLAYEAANYDMYRLKKLTLRYVPLVTVQNSGRV   73
6MRM_C_315        60 -PALFPTLAYEAANYDMYRLKKLTLRYVPLVTVQNSGRV   97
cons              79        :   .  :  :        : *       * :  117
```

```
1C8N_A_189     68 AMCLSYDRNDVAPGSRVQLSQTYKAINFPPYAGYDGAAI 106
1C8N_B_189     67 AMCLSYDRNDVAPGSRVQLSQTYKAINFPPYAGYDGAAI 105
1C8N_C_219     98 AMCLSYDRNDVAPGSRVQLSQTYKAINFPPYAGYDGAAI 136
1F2N_A_189     71 EMCFLYDYADTIPRYTGKMSRTAGFVTSSVWYGAEGCHL 109
1F2N_B_189     71 EMCFLYDYADTIPRYTGKMSRTAGFVTSSVWYGAEGCHL 109
1F2N_C_212     94 EMCFLYDYADTIPRYTGKMSRTAGFVTSSVWYGAEGCHL 132
1NG0_A_192     69 EMGFLLDYADSVPTNTRTMASSTSFTTSNVWGGGDGSSL 107
1NG0_B_192     69 EMGFLLDYADSVPTNTRTMASSTSFTTSNVWGGGDGSSL 107
1NG0_C_218     95 EMGFLLDYADSVPTNTRTMASSTSFTTSNVWGGGDGSSL 133
1OPO_A_267     71 ALGFNDDASDTPPTTKVGFYDLGKHVETAAQTAKDLVIP 109
1OPO_B_267     71 ALGFNDDASDTPPTTKVGFYDLGKHVETAAQTAKDLVIP 109
1OPO_C_268     72 ALGFNDDASDTPPTTKVGFYDLGKHVETAAQTAKDLVIP 110
1SMV_A_196     68 HMGFQYDMADTVPVSVNKLSNLRGYVSGQVWSGSAGLCF 106
1SMV_B_196     68 HMGFQYDMADTVPVSVNKLSNLRGYVSGQVWSGSAGLCF 106
1SMV_C_222     94 HMGFQYDMADTVPVSVNKLSNLRGYVSGQVWSGSAGLCF 132
2IZW_A_178     76 AMAMLYDVADVTSITIERLMQTRGGTWGPIWSPTRKR-- 112
2IZW_B_179     77 AMAMLYDVADVTSITIERLMQTRGGTWGPIWSPTRKR-- 113
2IZW_C_208    106 AMAMLYDVADVTSITIERLMQTRGGTWGPIWSPTRKR-- 142
2TBV_A_287     76 ALYFDKDSQDPEPADRVELANFGVLKETAPWAEAMLRIP 114
2TBV_B_286     76 ALYFDKDSQDPEPADRVELANFGVLKETAPWAEAMLRIP 114
2TBV_C_322    111 ALYFDKDSQDPEPADRVELANFGVLKETAPWAEAMLRIP 149
2ZAH_A_296     76 SILWDRDSQDPLPIDRAAISSYAHYADSAPWAENVLVVP 114
2ZAH_B_297     77 SILWDRDSQDPLPIDRAAISSYAHYADSAPWAENVLVVP 115
2ZAH_C_331    111 SILWDRDSQDPLPIDRAAISSYAHYADSAPWAENVLVVP 149
4LLF_A_290     79 ALYFDKDSEDPGPDDRAALANYAHLSEISPWAITKLTVP 117
4LLF_B_290     79 ALYFDKDSEDPGPDDRAALANYAHLSEISPWAITKLTVP 117
4LLF_B_292     81 ALYFDKDSEDPGPDDRAALANYAHLSEISPWAITKLTVP 119
4LLF_C_323    112 ALYFDKDSEDPGPDDRAALANYAHLSEISPWAITKLTVP 150
4SBV_A_199     71 HMGFQYDMADTLPVSVNQLSNLKGYVTGPVWEGQSGLCF 109
4SBV_B_199     71 HMGFQYDMADTLPVSVNQLSNLKGYVTGPVWEGQSGLCF 109
4SBV_C_222     94 HMGFQYDMADTLPVSVNQLSNLKGYVTGPVWEGQSGLCF 132
6MRL_A_295     79 SLFWDKDSQDPLPVDRAALSSYGHSNEGPPWAETTLNVP 117
6MRL_B_295     79 SLFWDKDSQDPLPVDRAALSSYGHSNEGPPWAETTLNVP 117
6MRL_C_326    110 SLFWDKDSQDPLPVDRAALSSYGHSNEGPPWAETTLNVP 148
6MRM_A_291     74 AMIWDPDSQDSAPQSRQEISAYSRSVSTAVYEKCSLTIP 112
6MRM_B_291     74 AMIWDPDSQDSAPQSRQEISAYSRSVSTAVYEKCSLTIP 112
6MRM_C_315     98 AMIWDPDSQDSAPQSRQEISAYSRSVSTAVYEKCSLTIP 136
cons          118  :    *  *  .     :                     156
```

```
1C8N_A_189      107 LNTDVTPTS--AIYVDVDVTRFDKAWYSTIGTAAFAALT 143
1C8N_B_189      106 LNTDVTPTS--AIYVDVDVTRFDKAWYSTIGTAAFAALT 142
1C8N_C_219      137 LNTDVTPTS--AIYVDVDVTRFDKAWYSTIGTAAFAALT 173
1F2N_A_189      110 LSG--GSARN-AVVASMDCSRVG--WKRVTSSIP----S 139
1F2N_B_189      110 LSG--GSARN-AVVASMDCSRVG--WKRVTSSIP----S 139
1F2N_C_212      133 LSG--GSARN-AVVASMDCSRVG--WKRVTSSIP----S 162
1NG0_A_192      108 LHTSMKSMGN-AVTSALPCDEFSNKWFKLSWSTPE---E 142
1NG0_B_192      108 LHTSMKSMGN-AVTSALPCDEFSNKWFKLSWSTPE---E 142
1NG0_C_218      134 LHTSMKSMGN-AVTSALPCDEFSNKWFKLSWSTPE---E 168
1OPO_A_267      110 VDGKTRFIRDSASDDAKLVDFGRIVLSTYGFDKADTVVG 148
1OPO_B_267      110 VDGKTRFIRDSASDDAKLVDFGRIVLSTYGFDKADTVVG 148
1OPO_C_268      111 VDGKTRFIRDSASDDAKLVDFGRIVLSTYGFDKADTVVG 149
1SMV_A_196      107 INNSRCSDTSTAISTTLDVSELGKKWYPYKTSADYATAV 145
1SMV_B_196      107 INNSRCSDTSTAISTTLDVSELGKKWYPYKTSADYATAV 145
1SMV_C_222      133 INNSRCSDTSTAISTTLDVSELGKKWYPYKTSADYATAV 171
2IZW_A_178      113 --------------LSYDPEHASLPWYLS---------- 127
2IZW_B_179      114 --------------LSYDPEHASLPWYLS---------- 128
2IZW_C_208      143 --------------LSYDPEHASLPWYLS---------- 157
2TBV_A_287      115 TDKVKRYCNDSATVDQKLIDLGQLGIATY-GGAGADAVG 152
2TBV_B_286      115 TDKVKRYCNDSATVDQKLIDLGQLGIATY-GGAGADAVG 152
2TBV_C_322      150 TDKVKRYCNDSATVDQKLIDLGQLGIATY-GGAGADAVG 187
2ZAH_A_296      115 CDNTWRYMNDTNAVDRKLVDFGQFLFATY-SGAGATAHG 152
2ZAH_B_297      116 CDNTWRYMNDTNAVDRKLVDFGQFLFATY-SGAGATAHG 153
2ZAH_C_331      150 CDNTWRYMNDTNAVDRKLVDFGQFLFATY-SGAGATAHG 187
4LLF_A_290      118 TDNVKRFISDTSSGDPKLINLGQFGWVAY-SGP-TAELG 154
4LLF_B_290      118 TDNVKRFISDTSSGDPKLINLGQFGWVAY-SGP-TAELG 154
4LLF_B_292      120 TDNVKRFISDTSSGDPKLINLGQFGWVAY-SGP-TAELG 156
4LLF_C_323      151 TDNVKRFISDTSSGDPKLINLGQFGWVAY-SGP-TAELG 187
4SBV_A_199      110 VNNTKCPDTSRAITIALDTNEVSEKRYPFKTATDYATAV 148
4SBV_B_199      110 VNNTKCPDTSRAITIALDTNEVSEKRYPFKTATDYATAV 148
4SBV_C_222      133 VNNTKCPDTSRAITIALDTNEVSEKRYPFKTATDYATAV 171
6MRL_A_295      118 TDGKQRFVTDSNTTDRKLVDLGQFAFATY-AGGSNNQIG 155
6MRL_B_295      118 TDGKQRFVTDSNTTDRKLVDLGQFAFATY-AGGSNNQIG 155
6MRL_C_326      149 TDGKQRFVTDSNTTDRKLVDLGQFAFATY-AGGSNNQIG 186
6MRM_A_291      113 ADNQWRFVADNTTVDRKLVDFGQLLFVTH-SGSDGIETG 150
6MRM_B_291      113 ADNQWRFVADNTTVDRKLVDFGQLLFVTH-SGSDGIETG 150
6MRM_C_315      137 ADNQWRFVADNTTVDRKLVDFGQLLFVTH-SGSDGIETG 174
cons            157                                         195
```

```
1C8N_A_189     144 AFDQ---NQFCPCTVHIGS-DGGPAVAV-----------  167
1C8N_B_189     143 AFDQ---NQFCPCTVHIGS-DGGPAVAV-----------  166
1C8N_C_219     174 AFDQ---NQFCPCTVHIGS-DGGPAVAV-----------  197
1F2N_A_189     140 SVDPNVVNTILPARLAVRS-SIKPTVSDT----------  167
1F2N_B_189     140 SVDPNVVNTILPARLAVRS-SIKPTVSDT----------  167
1F2N_C_212     163 SVDPNVVNTILPARLAVRS-SIKPTVSDT----------  190
1NG0_A_192     143 SENAHLTDTYVPARFVVRS-DFPVVTADQ----------  170
1NG0_B_192     143 SENAHLTDTYVPARFVVRS-DFPVVTADQ----------  170
1NG0_C_218     169 SENAHLTDTYVPARFVVRS-DFPVVTADQ----------  196
1OPO_A_267     149 ELFIQYTIVLSDPTKTAKISQASNDKVSD--------GP  179
1OPO_B_267     149 ELFIQYTIVLSDPTKTAKISQASNDKVSD--------GP  179
1OPO_C_268     150 ELFIQYTIVLSDPTKTAKISQASNDKVSD--------GP  180
1SMV_A_196     146 GVDVNIATDLVPARLVIALLDGSSSTAVA----------  174
1SMV_B_196     146 GVDVNIATDLVPARLVIALLDGSSSTAVA----------  174
1SMV_C_222     172 GVDVNIATDLVPARLVIALLDGSSSTAVA----------  200
2IZW_A_178     128 GVSSGAAAGNIQTPFQIAWAAQSSLVSTT----------  156
2IZW_B_179     129 GVSSGAAAGNIQTPFQIAWAAQSSLVSTT----------  157
2IZW_C_208     158 GVSSGAAAGNIQTPFQIAWAAQSSLVSTT----------  186
2TBV_A_287     153 ELFLARSVTLYFPQPTNTLLSSSKRLDLT-GSLADATGP  190
2TBV_B_286     153 ELFLARSVTLYFPQPTNTLLSS-KRLDLT-GSLADATGP  189
2TBV_C_322     188 ELFLARSVTLYFPQPTNTLLSSSKRLDLT-GSLADATGP  225
2ZAH_A_296     153 DLYVEYAVEFKDPQPIAGMVCMFDRLVSFSEVGSTIKGV  191
2ZAH_B_297     154 DLYVEYAVEFKDPQPIAGMVCMFDRLVSFSEVGSTIKGV  192
2ZAH_C_331     188 DLYVEYAVEFKDPQPIAGMVCMFDRLVSFSEVGSTIKGV  226
4LLF_A_290     155 DIFVEYTVDLFEAQPTSPLLESLFRESAS-SVQTRMGLP  192
4LLF_B_290     155 DIFVEYTVDLFEAQPTSPLLESLFRESAS-SVQTRMGLP  192
4LLF_B_292     157 DIFVEYTVDLFEAQPTSPLLESLFRESAS-SVQTRMGLP  194
4LLF_C_323     188 DIFVEYTVDLFEAQPTSPLLESLFRESAS-SVQTRMGLP  225
4SBV_A_199     149 GVNANIGNILVPARLVTAMEGGSSKTAVN----------  177
4SBV_B_199     149 GVNANIGNILVPARLVTAMEGGSSKTAVN----------  177
4SBV_C_222     172 GVNANIGNILVPARLVTAMEGGSSKTAVN----------  200
6MRL_A_295     156 DIYVEYGVEFSEAQPAGGLTQYITKSVG---ATASTTGP  191
6MRL_B_295     156 DIYVEYGVEFSEAQPAGGLTQYITKSVG---ATASTTGP  191
6MRL_C_326     187 DIYVEYGVEFSEAQPAGGLTQYITKSVG---ATASTTGP  222
6MRM_A_291     151 DIFLDCEVEFKGPQPTASIVQKTVIDLG--GTLTSFEGP  187
6MRM_B_291     151 DIFLDCEVEFKGPQPTASIVQKTVIDLG--GTLTSFEGP  187
6MRM_C_315     175 DIFLDCEVEFKGPQPTASIVQKTVIDLG--GTLTSFEGP  211
cons           196                                          234
```

```
1C8N_A_189    168 --------------------PPGDIFFKYVIELIEPINP 186
1C8N_B_189    167 --------------------PPGDIFFKYVIELIEPINP 185
1C8N_C_219    198 --------------------PPGDIFFKYVIELIEPINP 216
1F2N_A_189    168 ---------------------PGKLYVIASMVLRDPVDP 185
1F2N_B_189    168 ---------------------PGKLYVIASMVLRDPVDP 185
1F2N_C_212    191 ---------------------PGKLYVIASMVLRDPVDP 208
1NG0_A_192    171 ---------------------PGHLWLRSRILLKGSVSP 188
1NG0_B_192    171 ---------------------PGHLWLRSRILLKGSVSP 188
1NG0_C_218    197 ---------------------PGHLWLRSRILLKGSVSP 214
1OPO_A_267    180 TYVVPS---VNGNELQLRVVAAGKWCIIVRGT-VEGGFT 214
1OPO_B_267    180 TYVVPS---VNGNELQLRVVAAGKWCIIVRGT-VEGGFT 214
1OPO_C_268    181 TYVVPS---VNGNELQLRVVAAGKWCIIVRGT-VEGGFT 215
1SMV_A_196    175 ---------------------AGRIYDTYTIQMIEPTAS 192
1SMV_B_196    175 ---------------------AGRIYDTYTIQMIEPTAS 192
1SMV_C_222    201 ---------------------AGRIYDTYTIQMIEPTAS 218
2IZW_A_178    157 ---------------------LGRIMAEYLVELTDPVDV 174
2IZW_B_179    158 ---------------------LGRIMAEYLVELTDPVDV 175
2IZW_C_208    187 ---------------------LGRIMAEYLVELTDPVDV 204
2TBV_A_287    191 GYLVLT---RTPTVLTHTFRATGTFNLSGGLR-CLTS-L 224
2TBV_B_286    190 GYLVLT---RTPTVLTHTFRATGTFNLSGGLR-CLTS-L 223
2TBV_C_322    226 GYLVLT---RTPTVLTHTFRATGTFNLSGGLR-CLTS-L 259
2ZAH_A_296    192 NYIADRDVITTGGNIGVNINIPGTYLVTIVLNATSIGSL 230
2ZAH_B_297    193 NYIADRDVITTGGNIGVNINIPGTYLVTIVLNATSIGSL 231
2ZAH_C_331    227 NYIADRDVITTGGNIGVNINIPGTYLVTIVLNATSIGSL 265
4LLF_A_290    193 YFSLEV---ASATDLVWQARVPGTYVVTIIFN-STVGGL 227
4LLF_B_290    193 YFSLEV---ASATDLVWQARVPGTYVVTIIFN-STVGGL 227
4LLF_B_292    195 YFSLEV---ASATDLVWQARVPGTYVVTIIFN-STVGGL 229
4LLF_C_323    226 YFSLEV---ASATDLVWQARVPGTYVVTIIFN-STVGGL 260
4SBV_A_199    178 ---------------------TGRLYASYTIRLIEPIAA 195
4SBV_B_199    178 ---------------------TGRLYASYTIRLIEPIAA 195
4SBV_C_222    201 ---------------------TGRLYASYTIRLIEPIAA 218
6MRL_A_295    192 SYVVDANINVNATTANVEFFSPGTFLITAVVYGSTIASP 230
6MRL_B_295    192 SYVVDANINVNATTANVEFFSPGTFLITAVVYGSTIASP 230
6MRL_C_326    223 SYVVDANINVNATTANVEFFSPGTFLITAVVYGSTIASP 261
6MRM_A_291    188 SYLMPPDAFITSSSFGLFVDVAGTYLLTLVVTCSTTGSV 226
6MRM_B_291    188 SYLMPPDAFITSSSFGLFVDVAGTYLLTLVVTCSTTGSV 226
6MRM_C_315    212 SYLMPPDAFITSSSFGLFVDVAGTYLLTLVVTCSTTGSV 250
cons          235                       *                 273
```

```
1C8N_A_189    187 TMN------------------------------------ 189
1C8N_B_189    186 TMNV----------------------------------- 189
1C8N_C_219    217 TMN------------------------------------ 219
1F2N_A_189    186 TLNT----------------------------------- 189
1F2N_B_189    186 TLNT----------------------------------- 189
1F2N_C_212    209 TLNT----------------------------------- 212
1NG0_A_192    189 STNL----------------------------------- 192
1NG0_B_192    189 STNL----------------------------------- 192
1NG0_C_218    215 STNL----------------------------------- 218
1OPO_A_267    215 KPTLIGPGISGDVDYESARPIAVCELVTQMEGQILKITK 253
1OPO_B_267    215 KPTLIGPGISGDVDYESARPIAVCELVTQMEGQILKITK 253
1OPO_C_268    216 KPTLIGPGISGDVDYESARPIAVCELVTQMEGQILKITK 254
1SMV_A_196    193 ALNL----------------------------------- 196
1SMV_B_196    193 ALNL----------------------------------- 196
1SMV_C_222    219 ALNL----------------------------------- 222
2IZW_A_178    175 TINQ----------------------------------- 178
2IZW_B_179    176 TINQ----------------------------------- 179
2IZW_C_208    205 TINQ----------------------------------- 208
2TBV_A_287    225 TLGATGAVVINDILAIDNVGTASDYFLNCTVSSLPATVT 263
2TBV_B_286    224 TLGATGAVVINDILAIDNVGTASDYFLNCTVSSLPATVT 262
2TBV_C_322    260 TLGATGAVVINDILAIDNVGTASDYFLNCTVSSLPATVT 298
2ZAH_A_296    231 TFTG-NSKLVGNSLNVTSSGASALTFTLNSTGVPNSSNS 268
2ZAH_B_297    232 TFTG-NSKLVGNSLNVTSSGASALTFTLNSTGVPNSSNS 269
2ZAH_C_331    266 TFTG-NSKLVGNSLNVTSSGASALTFTLNSTGVPNSSNS 303
4LLF_A_290    228 TPSISGGGTINSSFSVSTAG-SSAYVANITIRVNANLSL 265
4LLF_B_290    228 TPSISGGGTINSSFSVSTAG-SSAYVANITIRVNANLSL 265
4LLF_B_292    230 TPSISGGGTINSSFSVSTAG-SSAYVANITIRVNANLSL 267
4LLF_C_323    261 TPSISGGGTINSSFSVSTAG-SSAYVANITIRVNANLSL 298
4SBV_A_199    196 ALNL----------------------------------- 199
4SBV_B_199    196 ALNL----------------------------------- 199
4SBV_C_222    219 ALNL----------------------------------- 222
6MRL_A_295    231 SMAGGNGTLIGDLPVVGGSNASIWTCVFSTTGVSTSVPT 269
6MRL_B_295    231 SMAGGNGTLIGDLPVVGGSNASIWTCVFSTTGVSTSVPT 269
6MRL_C_326    262 SMAGGNGTLIGDLPVVGGSNASIWTCVFSTTGVSTSVPT 300
6MRM_A_291    227 TVGG-NSTLVGDGRAAYGSSNYIASIVFTSSGVLSTTPS 264
6MRM_B_291    227 TVGG-NSTLVGDGRAAYGSSNYIASIVFTSSGVLSTTPS 264
6MRM_C_315    251 TVGG-NSTLVGDGRAAYGSSNYIASIVFTSSGVLSTTPS 288
cons          274                                         312
```

```
1C8N_A_189      190 ----------------------------- 189
1C8N_B_189      190 ----------------------------- 189
1C8N_C_219      220 ----------------------------- 219
1F2N_A_189      190 ----------------------------- 189
1F2N_B_189      190 ----------------------------- 189
1F2N_C_212      213 ----------------------------- 212
1NG0_A_192      193 ----------------------------- 192
1NG0_B_192      193 ----------------------------- 192
1NG0_C_218      219 ----------------------------- 218
1OPO_A_267      254 TSAEQP------LQWVVYRM--------- 267
1OPO_B_267      254 TSAEQP------LQWVVYRM--------- 267
1OPO_C_268      255 TSAEQP------LQWVVYRM--------- 268
1SMV_A_196      197 ----------------------------- 196
1SMV_B_196      197 ----------------------------- 196
1SMV_C_222      223 ----------------------------- 222
2IZW_A_178      179 ----------------------------- 178
2IZW_B_179      180 ----------------------------- 179
2IZW_C_208      209 ----------------------------- 208
2TBV_A_287      264 FTVSG----VAAGILLVGRARAN-VVNLL 287
2TBV_B_286      263 FTVSG----VAAGILLVGRARAN-VVNLL 286
2TBV_C_322      299 FTVSG----VAAGILLVGRARAN-VVNLL 322
2ZAH_A_296      269 SFSVGTVVALTRVRMTITRCSPETAYLA- 296
2ZAH_B_297      270 SFSVGTVVALTRVRMTITRCSPETAYLA- 297
2ZAH_C_331      304 SFSVGTVVALTRVRMTITRCSPETAYLA- 331
4LLF_A_290      266 SGLTG----ATNAQLFAVRAITENAVQVV 290
4LLF_B_290      266 SGLTG----ATNAQLFAVRAITENAVQVV 290
4LLF_B_292      268 SGLTG----ATNAQLFAVRAITENAVQVV 292
4LLF_C_323      299 SGLTG----ATNAQLFAVRAITENAVQVV 323
4SBV_A_199      200 ----------------------------- 199
4SBV_B_199      200 ----------------------------- 199
4SBV_C_222      223 ----------------------------- 222
6MRL_A_295      270 FTQAG--TGLTRVQYTITRVNSQTAYQV- 295
6MRL_B_295      270 FTQAG--TGLTRVQYTITRVNSQTAYQV- 295
6MRL_C_326      301 FTQAG--TGLTRVQYTITRVNSQTAYQV- 326
6MRM_A_291      265 VQFSGS-SGVSRVQMNICRCKQGNTFIL- 291
6MRM_B_291      265 VQFSGS-SGVSRVQMNICRCKQGNTFIL- 291
6MRM_C_315      289 VQFSGS-SGVSRVQMNICRCKQGNTFIL- 315
cons            313                               341
```

BAD AVG GOOD

Data S4: MSA of A, B and C chains from the IAUs of all *Tymoviridae* structures.

```
1AUY_A_163      1 ---------------------------SPLTIKQPFQSEV  13
1AUY_B_189      1 MEIDKELAPQDRTVTVATVLPAVPGPSPLTIKQPFQSEV  39
1AUY_C_189      1 MEIDKELAPQDRTVTVATVLPAVPGPSPLTIKQPFQSEV  39
1DDL_A_190      1 MEQDKILAHQASLNTKPSLLPPPVGNPPPVISYPFQITL  39
1DDL_B_191      1 MEQDKILAHQASLNTKPSLLPPPVGNPPPVISYPFQITL  39
1DDL_C_175      1 -------------NTKPSLLPPPVGNPPPVISYPFQITL  26
1E57_A_160      1 ---------------------------SPAIVLPFQFEA  12
1E57_B_183      1 ----VVKVKQASIPAPGSILSQPNTEQSPAIVLPFQFEA  35
1E57_C_179      1 --------KQASIPAPGSILSQPNTEQSPAIVLPFQFEA  31
cons            1                            . .*  ***     39

1AUY_A_163     14 LFAGTKDAEASLTIANIDSVSTLTTFYRHASLESLWVTI  52
1AUY_B_189     40 LFAGTKDAEASLTIANIDSVSTLTTFYRHASLESLWVTI  78
1AUY_C_189     40 LFAGTKDAEASLTIANIDSVSTLTTFYRHASLESLWVTI  78
1DDL_A_190     40 ASLGTEDAADSVSIASNSVLATYTALYRHAQLKHLKATI  78
1DDL_B_191     40 ASLGTEDAADSVSIASNSVLATYTALYRHAQLKHLKATI  78
1DDL_C_175     27 ASLGTEDAADSVSIASNSVLATYTALYRHAQLKHLKATI  65
1E57_A_160     13 TTFGTAETAAQVSLQTADPITKLTAPYRHAQIVECKAIL  51
1E57_B_183     36 TTFGTAETAAQVSLQTADPITKLTAPYRHAQIVECKAIL  74
1E57_C_179     32 TTFGTAETAAQVSLQTADPITKLTAPYRHAQIVECKAIL  70
cons           40    ** ::  .::: . . ::. *: ****.:    . :  78

1AUY_A_163     53 HPTLQAPTFPTTVGVCWVPAQSPVTPAQITKTYGGQIFC  91
1AUY_B_189     79 HPTLQAPTFPTTVGVCWVPAQSPVTPAQITKTYGGQIFC 117
1AUY_C_189     79 HPTLQAPTFPTTVGVCWVPAQSPVTPAQITKTYGGQIFC 117
1DDL_A_190     79 HPTYMAPKYPTSVALVWVPANSTATSTQVLDTYGGLHFC 117
1DDL_B_191     79 HPTYMAPKYPTSVALVWVPANSTATSTQVLDTYGGLHFC 117
1DDL_C_175     66 HPTYMAPKYPTSVALVWVPANSTATSTQVLDTYGGLHFC 104
1E57_A_160     52 TPTDLAVSNPLTVYLAWVPANSPATPTQILRVYGGQSFV  90
1E57_B_183     75 TPTDLAVSNPLTVYLAWVPANSPATPTQILRVYGGQSFV 113
1E57_C_179     71 TPTDLAVSNPLTVYLAWVPANSPATPTQILRVYGGQSFV 109
cons           79  **  * . * :* : ****:*..*.:*:  .***  *  117

1AUY_A_163     92 IGGAIQTLSPLIVKCPLEMMQPRVKDSIQYLDSPKLLIS 130
1AUY_B_189    118 IGGAIQTLSPLIVKCPLEMMQPRVKDSIQYLDSPKLLIS 156
1AUY_C_189    118 IGGAIQTLSPLIVKCPLEMMQPRVKDSIQYLDSPKLLIS 156
1DDL_A_190    118 IGGSVNSVKPIDVEANLTNLNPIIKASTTFTDTPKLLYY 156
1DDL_B_191    118 IGGSVNSVKPIDVEANLTNLNPIIKASTTFTDTPKLLYY 156
1DDL_C_175    105 IGGSVNSVKPIDVEANLTNLNPIIKASTTFTDTPKLLYY 143
1E57_A_160     91 LGGAISAAKTIEVPLNLDSVNRMLKDSVTYTDTPKLLAY 129
1E57_B_183    114 LGGAISAAKTIEVPLNLDSVNRMLKDSVTYTDTPKLLAY 152
1E57_C_179    110 LGGAISAAKTIEVPLNLDSVNRMLKDSVTYTDTPKLLAY 148
cons          118 :**::.: ..: *   *  ::  :* *  : *:****   156
```

```
1AUY_A_163     131 ITAQPTAPPASTCIITVSGTLSMHSPLITDTST--  163
1AUY_B_189     157 ITAQPTAPPASTCIITVSGTLSMHSPLITDTST--  189
1AUY_C_189     157 ITAQPTAPPASTCIITVSGTLSMHSPLITDTST--  189
1DDL_A_190     157 SKAQATAPTSPTCYLTIQGQIELSSPLLQASSSS-  190
1DDL_B_191     157 SKAQATAPTSPTCYLTIQGQIELSSPLLQAASSSS  191
1DDL_C_175     144 SKAQATAPTSPTCYLTIQGQIELSSPLLQASS---  175
1E57_A_160     130 SRAPTNPSKIPTASIQISGRIRLSKPMLIAN----  160
1E57_B_183     153 SRAPTNPSKIPTASIQISGRIRLSKPMLIAN----  183
1E57_C_179     149 SRAPTNPSKIPTASIQISGRIRLSKPMLIAN----  179
cons           157   * ....  .*. : :.* : : .*::         191

BAD AVG GOOD
```

SUPPLEMENTARY REFERENCES

CASPAR, D.L., KLUG, A., 1962. Physical principles in the construction of regular viruses. Cold Spring Harb Symp Quant Biol. https://doi.org/10.1101/SQB.1962.027.001.005

Clementi, C., Nymeyer, H., Onuchic, J.N., 2000. Topological and energetic factors: what determines the structural details of the transition state ensemble and “en-route” intermediates for protein folding? an investigation for small globular proteins. J Mol Biol 298, 937–953. https://doi.org/10.1006/jmbi.2000.3693

di Tommaso, P., Moretti, S., Xenarios, I., Orobitg, M., Montanyola, A., Chang, J.M., Taly, J.F., Notredame, C., 2011. T-Coffee: A web server for the multiple sequence alignment of protein and RNA sequences using structural information and homology extension. Nucleic Acids Res 39. https://doi.org/10.1093/nar/gkr245

le Guilloux, V., Schmidtke, P., Tuffery, P., 2009. Fpocket: An open source platform for ligand pocket detection. BMC Bioinformatics 10. https://doi.org/10.1186/1471-2105-10-168

Meng, E.C., Pettersen, E.F., Couch, G.S., Huang, C.C., Ferrin, T.E., 2006. Tools for integrated sequence-structure analysis with UCSF Chimera. BMC Bioinformatics 7, 339. https://doi.org/10.1186/1471-2105-7-339

Noel, J.K., Levi, M., Raghunathan, M., Lammert, H., Hayes, R.L., Onuchic, J.N., Whitford, P.C., 2016. SMOG 2: A Versatile Software Package for Generating Structure-Based Models. PLoS Comput Biol 12, e1004794. https://doi.org/10.1371/journal.pcbi.1004794

Onuchic, J.N., Luthey-Schulten, Z., Wolynes, P.G., 1997. THEORY OF PROTEIN FOLDING: The Energy Landscape Perspective. Annu Rev Phys Chem 48, 545–600. https://doi.org/10.1146/annurev.physchem.48.1.545

Onuchic, J.N., Wolynes, P.G., 2004. Theory of protein folding. Curr Opin Struct Biol 14, 70–75. https://doi.org/10.1016/j.sbi.2004.01.009

Pettersen, E.F., Goddard, T.D., Huang, C.C., Couch, G.S., Greenblatt, D.M., Meng, E.C., Ferrin, T.E., 2004. UCSF Chimera - A visualization system for exploratory research and analysis. J Comput Chem 25, 1605–1612. https://doi.org/10.1002/jcc.20084

Prakash, D.L., Gosavi, S., 2021. Understanding the Folding Mediated Assembly of the Bacteriophage MS2 Coat Protein Dimers. Journal of Physical Chemistry B 125, 8722–8732. https://doi.org/10.1021/ACS.JPCB.1C03928/SUPPL_FILE/JP1C03928_SI_001.PDF

Sobolev, V., Sorokine, A., Prilusky, J., Abola, E.E., Edelman, M., 1999. Automated analysis of interatomic contacts in proteins. Bioinformatics 15, 327–332. https://doi.org/10.1093/bioinformatics/15.4.327

Thompson, J.D., Gibson, Toby.J., Higgins, D.G., 2003. Multiple Sequence Alignment Using ClustalW and ClustalX. Curr Protoc Bioinformatics 00. https://doi.org/10.1002/0471250953.bi0203s00

van der Spoel, D., Lindahl, E., Hess, B., Groenhof, G., Mark, A.E., Berendsen, H.J.C., 2005. GROMACS: Fast, flexible, and free. J Comput Chem 26, 1701–1718. https://doi.org/10.1002/jcc.20291
